# Lonelier people feel less empathic despite intact neural empathy responses after meditation training

**DOI:** 10.1093/scan/nsag015

**Published:** 2026-06-20

**Authors:** Marla Dressel, Naomi Nero, Paige Freeburg, Melinda C Somers, Joseph S Venticinque, Ashley S VanMeter, Shawn A Rhoads, Abigail A Marsh

**Affiliations:** Department of Psychology, Georgetown University, Washington, D.C., DC 20057, United States; Department of Psychology, Georgetown University, Washington, D.C., DC 20057, United States; Department of Psychology, Yale University, New Haven, CT 06510, United States; Department of Psychology, Georgetown University, Washington, D.C., DC 20057, United States; Department of Psychology, Georgetown University, Washington, D.C., DC 20057, United States; Department of Neurology, Georgetown University Medical Center, Washington, D.C., DC 20007, United States; Department of Psychiatry, Icahn School of Medicine at Mount Sinai, New York, NY 10029, United States; Nash Family Department of Neuroscience, Icahn School of Medicine at Mount Sinai, New York, NY 10029, United States; Friedman Brain Institute, Icahn School of Medicine at Mount Sinai, New York, NY 10029, United States; Center for Computational Psychiatry, Icahn School of Medicine at Mount Sinai, New York, NY 10029, United States; Department of Psychology, Georgetown University, Washington, D.C., DC 20057, United States

**Keywords:** loneliness, empathy, meditation, multi-voxel pattern analysis, functional magnetic resonance imaging

## Abstract

Loneliness, which has reached an all-time high in the United States, has been linked to reduced self-reported empathy. Loving kindness meditation (LKM) is aimed at extending love and kindness to others and has been shown to increase empathy. But whether LKM can reduce loneliness, and whether this corresponds to higher levels of trait empathy, state empathy, and/or neural empathic responding, has not been assessed. In this pre-registered mixed-design randomized controlled trial, 108 participants completed LKM or active control training. Loneliness and trait empathy were assessed pre- and post-intervention. Neural empathic responding was measured in 54 participants during functional MRI by computing the multi-voxel pattern similarity between experiencing and observing both pain and fearful anticipation of pain. Both interventions reduced loneliness, but not trait empathy, which failed to support the hypothesis that LKM is effective in reducing loneliness by increasing empathy. Furthermore, we found no credible evidence that loneliness is associated with differences in neural empathic responding. However, loneliness was associated with lower self-reported empathy. Together, these results suggest that lonelier individuals simulate others’ experiences but may not subjectively perceive themselves as empathetic, emphasizing the potential of interventions that address maladaptive social cognition in loneliness.

## Introduction

Social isolation and loneliness have reached an all-time high in the United States, with nearly half of US adults reporting sometimes or always feeling lonely (Office of the U.S. Surgeon General 2023). Loneliness is defined not by how many social relationships a person has but by the quality of their relationships and their subjective experiences during social interactions (Cacioppo and Patrick 2008). It is linked to negative perceptions of interpersonal relationships (Lemay *et al.* 2024) and poor relationship quality (Wittenberg and Reis 1986), raising questions about the role of empathy in loneliness, given that empathy is important for sustaining high-quality social relationships (Kardos *et al*. 2017, Morelli *et al*. 2017, Elmi and Clapp 2021). Preliminary evidence linking loneliness and self-reported empathy suggests the potential utility of loneliness interventions that target empathy (Beadle *et al*. 2012, Lee *et al*. 2019, Hu *et al*. 2020). Loving kindness meditation (LKM), which is aimed at extending feelings of love and kindness to distant others (Salzberg and Kabat-zinn 2004), has been shown to increase empathy and related helping behaviors like generosity (Kok and Singer 2017, Kreplin *et al*. 2018). But whether LKM can reduce loneliness is not known, nor are the proximal mechanisms by which it may promote prosocial outcomes like empathy. Thus, we sought to test whether LKM can increase empathy and reduce loneliness. We considered three metrics of empathy—including trait empathy, state empathy, and neural empathic responding measured using functional magnetic resonance imaging (fMRI)—and assessed their association with loneliness following an LKM training intervention administered in a randomized controlled trial (RCT).

Because loneliness can negatively impact mental and physical health (OSG 2023), identifying effective interventions for reducing loneliness is essential for promoting societal well-being. A previous meta-analysis found the most effective loneliness interventions target social cognition rather than merely increasing opportunities for social contact (Masi *et al*. 2011), perhaps because miscalibrated social cognition can inhibit socially connecting with others (Epley *et al*. 2022). For example, lonelier people tend to misperceive features of their social interactions and social partners (Wittenberg and Reis 1986), including others’ empathy toward them (Pei *et al*. 2025). Moreover, despite forming relationships, lonelier people self-report fewer adaptive interpersonal skills necessary to maintain relationships (Wittenberg and Reis 1986, Lee *et al*. 2001). Thus, loneliness appears to reflect subjective perceptions of difficulties connecting with others.

Empathy, the ability to share and understand others’ emotions (Marsh 2018, Weisz and Cikara 2021), is a promising candidate for increasing social connectedness and reducing loneliness, particularly because it has been shown to be malleable through intervention (Weisz and Zaki 2017). Empathy is important for maintaining high-quality social relationships (Kardos *et al*. 2017, Morelli *et al*. 2017, Elmi and Clapp 2021) and is associated with enjoyment of social interactions (Gould and MacNeil Gautreau 2014) and perceptions of social closeness (Brethel-Haurwitz *et al*. 2018, Pearce *et al*. 2019). Numerous studies have found inverse correlations between loneliness and reduced self-reported empathy, even after accounting for gender, age, negative emotionality, and other potential covariates (Beadle *et al*. 2012, Lee *et al*. 2019, Hu *et al*. 2020). But because current studies linking empathy and loneliness are correlational, the causal relationship between them (which could be bidirectional) remains uncertain.

Interestingly, some studies find that lonelier individuals show comparable or even superior empathic abilities in specific domains (Mwilambwe-Tshilobo *et al*. 2023). For example, lonelier people have shown greater empathic accuracy (accurate recognition) for fearful, but not other, facial expressions (Di Tella *et al*. 2023). Discrepancies across studies may stem from the disparate ways in which empathy is defined and measured across investigators and disciplines (Hall and Schwartz 2019, Quesque *et al*. 2024). Distinctions are often drawn between dissociable processes like cognitive empathy, emotional empathy, and empathic concern (EC; also termed care, compassion, or sympathy), but how these terms are defined and operationalized varies widely (Marsh 2018). For example, some define “cognitive empathy” as the ability to understand cognitive states like intentions and beliefs (Blair 2005, Shamay-Tsoory 2011); others define it as the ability to verbally describe others’ sensory, affective, or cognitive states (Van Zonneveld *et al*. 2017). These inconsistencies may result from the practice of measuring empathy via self-report using a variety of scales that emphasize different constructs, are at most moderately inter-correlated (Lawrence *et al*. 2004, Bernardo *et al*. 2018, Innamorati *et al*. 2019), and often have only weak predictive validity in interpersonal settings (Marsh *et al*. 2007, Vachon *et al*. 2014, Bernardo *et al*. 2018).

Beyond theoretical decomposition of empathy into dissociable components (which in daily life typically co-occur, Depow and Inzlicht 2025 ), empathy can also be operationalized and measured at different levels, including at the trait, state, and neural level. Trait empathy reflects relatively stable individual differences in empathic responding across contexts, whereas state empathy captures momentary empathic responses in a specific context (Cameron 2018, Kozakevich Arbel *et al*. 2021). Both trait and state empathy are typically measured using self-report, with mixed correspondence between measures (Hall and Schwartz, 2019).

At the neural level, empathy is commonly operationalized as self—other neural mapping when experiencing and observing a specific state such as pain or fear. Specifically, shared neural representations between self and other have been argued to “lie at the core of the phenomena of empathy and affective sharing” (Lamm *et al*. 2011, p. 2492). Evidence for such mapping comes from univariate analyses showing shared regional engagement during experienced and observed emotions, as well as from multivariate pattern analyses demonstrating similarity in distributed neural response patterns across experienced and observed states (Lamm *et al*. 2011, Brethel-Haurwitz *et al*. 2018, Marsh 2018, O’Connell *et al*. 2019). Although the majority of neuroimaging research on empathy focuses on empathy for pain, empathy represents, by definition, an attempted simulation of any internal state experienced by another individual (Lamm *et al*. 2019, Marsh 2022). An extensive body of work in clinical populations has demonstrated that people vary in their tendency and capacity to empathize with distinct states, such as pain or fear (Bird and Viding 2014, Marsh 2018, O’Connell *et al*. 2019). Empathic self-other pattern similarity (which we will refer to as “neural empathy”) supersedes many limitations of self-reported empathy and is a conceptually transparent metric that can be objectively measured, does not rely on self-knowledge, and may better predict interpersonal outcomes, including perceived social connectedness (Hein *et al*. 2010, Levy *et al*. 2016, Brethel-Haurwitz *et al*. 2018). For example, multi-voxel pattern similarity and cross-classification to measure self-other neural mapping during experienced and observed pain in regions including the anterior midcingulate cortex (aMCC) and anterior insula (AI) predicts individual variation in prosocial behavior and low levels of traits like callousness better than self-reported empathy does (Hein *et al*. 2010, Brethel-Haurwitz *et al*. 2018, O’Connell *et al*. 2019, Berluti *et al*. 2020).

Supporting the potential importance of considering neural empathy in loneliness, structural and resting-state functional neuroimaging studies suggest loneliness is associated with anomalies in regions or connectivity between regions like the AI, anterior cingulate cortex (ACC), and superior temporal sulcus that are also associated with empathy (Kanai *et al*. 2012, Nakagawa *et al*. 2015, Layden *et al*. 2017, Düzel *et al*. 2019, Feng *et al*. 2019, Spreng *et al*. 2020, Schurz *et al*. 2021, Zajner *et al*. 2021). However, little research has been conducted on task-based functional neural activation patterns pertaining to loneliness, and much of what has been done focuses on social motivation, perceptions of others’ traits, or social reward rather than on others’ experiences (Cacioppo *et al*. 2009a, 2009b, Inagaki *et al*. 2016, Courtney and Meyer 2020, Tomova *et al*. 2021). It thus remains unclear whether and how loneliness is linked to neural empathy.

Several studies have now demonstrated that LKM training can increase self-reported empathy in adults (Wallmark *et al*. 2013, Leppma and Young 2016, Csaszar *et al*. 2018, Chen *et al*. 2021). A recent meta-analysis on prosocial outcomes of meditation found that increased self-reported empathy and compassion were among the most reliably observed outcomes following RCTs of meditation training, like LKM (Kreplin *et al*. 2018, but note most compassion studies assessed self-compassion). Prior findings that self-reported empathy is positively correlated with social connectedness and negatively correlated with loneliness (Beadle *et al*. 2012, Lee *et al*. 2019, Hu *et al*. 2020) support the possibility that LKM, by increasing empathy, can also promote social connectedness and reduce loneliness.

The present study used a pre-registered, mixed-design RCT to test the hypotheses that LKM (compared to an active control condition) would increase social connectedness and reduce loneliness and that any effects would be due to increases in empathy. We tested the self-reported trait empathy subscales empathic concern (EC) and perspective taking (PT; Davis 1983), which are typically interpreted as capturing the most prosocial forms of empathy (Tusche *et al*. 2016, Böckler *et al*. 2018, Weisz and Cikara 2021), self-reported state empathy (of pain, fear, and unpleasantness), and neural empathy (pattern similarity). We also considered associations among these measures to better understand how various measures of empathy are interrelated and how they correspond with experienced loneliness (see preregistration details: https://osf.io/2sx3v/).

## Materials and methods

### Participants

**Figure 1:**
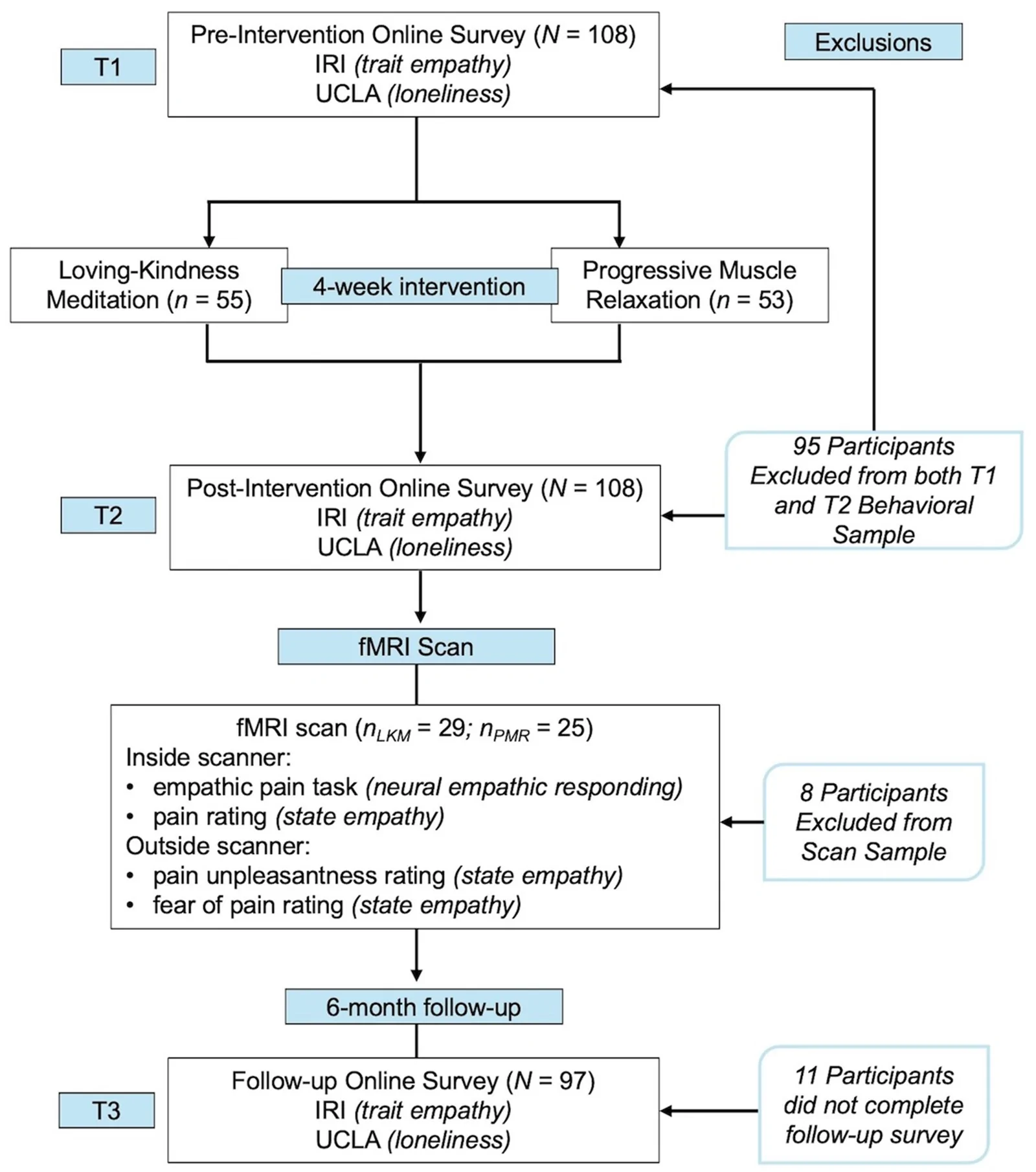
Study timeline. *Note*. Data were collected before the intervention (Time 1; T1), after completing the 4-week intervention (Time 2; T2), and 6 months after completing the intervention (Time 3; T3). At each time point (Time 1–Time 3), Trait Empathy [Interpersonal Reactivity Index (IRI; Davis 1983)] and Loneliness (UCLA v3; Russel 1996) were measured. Just after the intervention, 54 participants (29 LKM, 25 PMR) completed the fMRI scanning session, during which we assessed two additional measures of empathy: neural empathy (multi-voxel pattern similarity) using the empathic pain task and self-reported state empathy. State empathy for pain was reported verbally and based on the similarity of the participants’ rating of their own and their study partner’s pain after each run inside the scanner. State empathy for fear and unpleasantness were reported after the scan in a survey and were based on the similarity of the participant’s fear rating during anticipation and pain unpleasantness during painful stimulation for themselves and their study partner, respectively. In addition to noted exclusions in the figure, we provide specifics in Supplemental Information A.

Participants from the Washington, DC, area were recruited to be demographically representative of adults in the region in terms of age, gender, and ethnicity (Table 1). 108 participants completed the study, including 55 who completed LKM training and 53 demographically matched adults who completed progressive muscle relaxation (PMR), which was chosen as an active control intervention for its non-social nature. Online screening measures included demographics, prior meditation experience, MRI contraindications, and self-report Time 1 measures, including the UCLA Loneliness Scale-Version 3 (UCLA v3; Russell 1996), the Social Connectedness Scale Revised (SCS-R; Lee *et al*. 2001), the Inclusion of Other in the Self Scale (IOS; Aron *et al*. 1992), and the Interpersonal Reactivity Index assessing trait empathy (IRI; Davis 1983). All participants then completed 4 weeks of online LKM or PMR training. Fifty four participants (29 in the LKM group, 25 in the PMR group) also completed on-site testing, which included fMRI scanning as well as post-scan assessments. Screened participants were excluded for being less than 18 years old, non-fluency in English, prior experience with LKM or compassion meditation, current substance dependence or misuse, contraindications to safe MRI scanning, or failure to complete at least 75% of meditation training sessions (see online Supplementary Information A for full exclusion details). There were no statistically significant differences between intervention groups for any demographic characteristics (Table 1). Participants who completed all components of the study were compensated $220; participants who completed only behavioral testing received $120. Procedures were approved by the Institutional Review Board at Georgetown University, and all participants provided signed informed consent. See Fig. 1 for study design details.

**Table 1:** Sociodemographic characteristics of participants at baseline.

	Full sample (*n* = 108)	fMRI subsample (*n* = 54)	*P*	Effect size^a^
Age	Mean = 40.22 [37.10, 43.34]	Mean = 37.07 [34.43, 39.71]	.128	0.21 [−0.06, 0.48]
Woman^b^	65 (60.2%)	27 (50.0%)	.306	0.19 [−0.15, −.52]
Asian	20 (18.5%)	11 (20.4%)	.944	−0.05 [0.47, 0.36]
Black	15 (13.9%)	6 (11.1%)	.804	0.11 [−0.36, 0.58]
Hispanic/Latino	15 (11.1%)	8 (14.8%)	.673	−0.16 [−0.67, 0.36]
White	57 (52.8%)	27 (50%)	.868	0.52 [−0.27, 0.37]
Other	4 (3.7%)	2 (3.7%)	1	0 [−0.78, 0.78]
Right-handed	90 (83.3%)	45 (83.3%)	.999	0.03 [−0.42, 0.48]
Income ≥$60,000	78 (72.2%)	41 (75.9%)	.812	−0.09 [−0.48, 0.31]
≥Bachelor’s degree	83 (76.9%)	42 (77.8%)	.999	−0.02 [−0.39, 0.35]

*Note.* There were no statistically significant differences between the full sample and the fMRI sample in any demographic characteristics. For age, 95% confidence intervals are given in brackets.

a Effect sizes for gender, ethnicity, handedness, income, and education are *Cohen’s h*; effect size for age is *Cohen’s d*. For all tests, 95% confidence intervals are provided

b For gender, three participants reported “Non-binary”; for income, seven participants reported “Don’t know”; for handedness, three participants reported “Both”. These were excluded when calculating frequencies and testing for differences.

### Meditation training

LKM sessions used a similar approach as in previous studies (Brethel-Haurwitz *et al*. 2018, Rhoads *et al*. 2023) and were pre-recorded by renowned LKM expert Sharon Salzberg (https://www.sharonsalzberg.com). Sessions were aimed at progressively expanding feelings of closeness with the self, a benefactor, a neutral person, a friend, a distant friend, a difficult person, a group of individuals, and all beings (the focus of the LKM changed every three sessions). Pre-recorded PMR sessions (read by a female narrator) involved focusing on a different part of the body every three sessions, including hands and arms, face, head, neck and shoulders, abdomen and buttocks, legs and feet, all upper body, all lower body, and the whole body. No background music was included in any of the LKM or PMR sessions. The pre-recorded guided LKM or PMR sessions were sent via email 6 days per week (Monday through Saturday) at a time preferred by the participant and lasted 15 min per day. In total, participants completed 360 min of LKM or PMR training. The first day also involved listening to additional pre-recorded instructions (LKM: 12 min, PMR: 4 min). Researchers contacted participants with reminders if they missed a session. Attrition levels were <5%. All meditation materials were made available to all participants at the study’s conclusion.

### Self-report measures

Self-report measures were collected prior to LKM or PMR (Time 1), immediately after completion of LKM or PMR (Time 2), and 6 months after completion of LKM or PMR (Time 3) (Fig. 1). Key measures included the 20-item UCLA Loneliness Scale-Version 3 (UCLA v3; Russell 1996) (Cronbach’s alpha = .89 to .94; Russell 1996), the Social Connectedness Scale-Revised (Lee *et al*. 2001) (Cronbach’s alpha = .92; Lee *et al*. 2001), and the Interpersonal Reactivity Index (Davis 1983) (Cronbach’s alpha = .71 to .77), from which we focused on the empathic concern (EC) and perspective-taking (PT) subscales, which capture the most prosocial forms of empathy (Tusche *et al*. 2016, Böckler *et al*. 2018, Weisz and Cikara 2021). Additional measures included the Inclusion of Other in the Self Scale (IOS; Aron *et al*. 1992) (Cronbach’s alpha = .93; Aron *et al*. 1992) and the Cantril Ladder measure of subjective well-being (Cantril 1965).

### Empathic pain fMRI task

**Figure 2:**
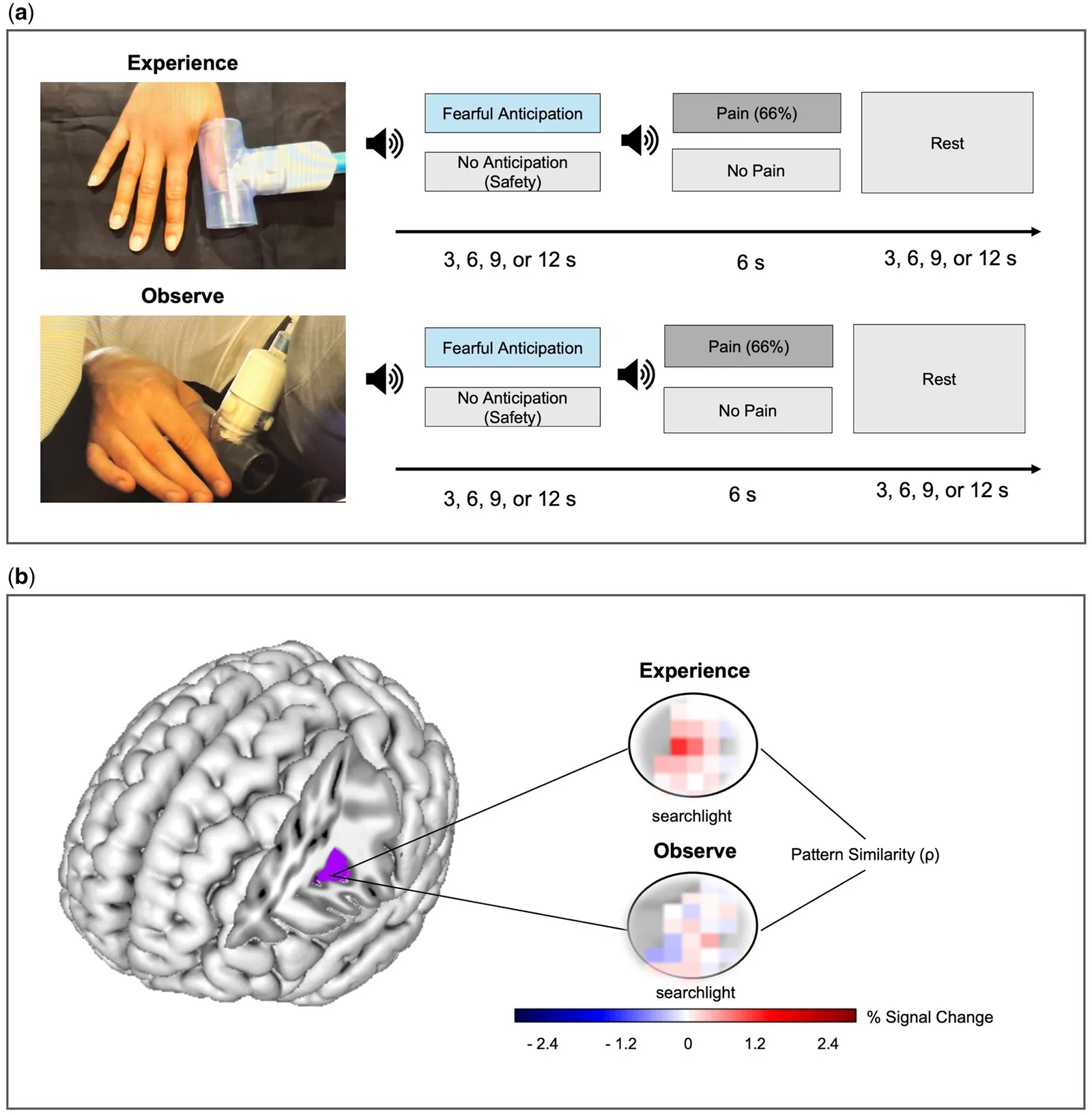
Empathic pain fMRI task design and pattern similarity analysis methodology. *Note*. (a) During each trial, participants heard anticipatory tone cues signaling that pain might or will not ensue, followed by experiences of pain (or no pain) and a rest period. Sixty-six percent of the time a pain cue occurred, participants would receive pressure pain to their right hand. Each experience and observe run thus consisted of pain trials, no pain trials, fearful anticipation trials, and safety trials. (b) A single subject’s pattern similarity (pain) in the AI and dACC. To assess pattern similarity (pain), we constructed three regions of interest consisting of the left and right AI and the dACC. Using a searchlight approach, we calculated the Spearman correlation for each subject of the voxel-wise patterns for experiencing pain vs no pain and observing pain vs no pain. The example shows these methods in the left AI.

The empathic pain task employed the same methods and apparatus used in prior work (Brethel-Haurwitz *et al*. 2018, O’Connell *et al*. 2019, Berluti *et al*. 2020, Vekaria *et al*. 2020). Thumbnail pressure–pain stimulation subjectively rated as “slightly intense” pain for each participant was applied to the base of the participant’s right thumbnail using a plastic rubber probe and a pneumatic device controlled in the console room outside the scanner. Upon entering the console room, participants met a female stranger described as their study partner who was actually a trained confederate and who did not converse with participants. The study partner was visibly connected to the thumbnail pressure–pain stimulus device in the console room at the time of introduction. Participants were informed that they would view their study partner’s hand through a live video feed. During fMRI scanning, participants viewed live video feeds of their own hand and the hand of their study partner receiving pressure pain using this device in two separate runs. Both hands were shown from similar angles against a black cloth background (Fig. 2a). Thus, the application of pain was clearly visible, including the movement of the stimulation device of the confederate’s hand, such that the participants did not need to infer whether pain was applied based on the auditory cue alone (see below for details on the auditory cues). We designed the study this way to facilitate accurate perception of the other’s experience and to make the self-pain and other-pain trials as similar as possible.

The experiment consisted of one run in which participants anticipated (fear trials) and experienced (pain trials) pain and one in which they observed their partner anticipating and experiencing pain (Fig. 2a). This design, in which fear trials are modeled separately, is consistent with the definition of fear as a multimodal state that accompanies the anticipation of an aversive outcome such as pain (O’Connell *et al*. 2024). Each trial lasted 24 s, beginning with a 3-s cue period, which started with a 1-s auditory cue and indicated whether the participant or their partner would potentially receive a painful stimulus (fear trial) or not (safety trial). This was followed by an anticipation period varying randomly in a jittered manner between 3, 6, 9, or 12 s (7.44 s on average), allowing separate analyses of pain and fear trials. The anticipation phase was followed by a 1-s auditory cue that indicated whether the participant was currently receiving painful stimulation or not and a 6-s period of constant moderately painful stimulation or no stimulation. Each run comprised 15 safety trials and 15 pain trials. One-third of pain trials omitted the pain stimulus to make the pain cues probabilistic, which effectively promotes fearful anticipation (Sylvers *et al*. 2011). The order of the runs was counterbalanced to mitigate order effects. Following scanning, participants were asked whether they believed their study partner was real and receiving stimulation in real time (mean belief rating: 5.57/7, SD = 1.71).

### Subjective ratings and state empathy

Following each run and while still in the scanner, participants rated their perceived pain experience from 1 (No pain) to 7 (Extreme pain). Immediately after completion of the scan in a behavioral testing room, participants retrospectively rated the unpleasantness of pain trials and fear/anxiety during the fear trials (1: Not at all to 7: Extremely). Participants also answered these regarding the experiences of their partner. These measures were used to assess state empathy, operationalized as how similarly participants rated their own experiences to those of their partner ((−1)* the absolute difference between the experience of the study partner and the experience of the subject) (Figure 1, see online supplementary material for a color version of this figure). By incorporating the self-rating, the difference score more directly captures how much the participant’s own state anchors their representation of the other’s state. Using this behavioral measure, smaller discrepancies between experienced and observed pain indicate more affective alignment between self and other, consistent with simulation and, consequently, higher state empathy.

### Statistical analyses of behavioral data

Analysis of behavioral measures was conducted in R (R Core Team 2021) and RStudio (Posit, PBC 2020). Perceived loneliness (UCLA v3; Russell 1996), social connectedness (Lee *et al*. 2001), trait empathy (IRI EC and PT scores respectively; Davis 1983), and inclusion of other in the self (IOS; Aron *et al*. 1992) were averaged for each subject. We estimated linear mixed-effects models by fitting the maximal random effects structure of within-participant factors subsumed by each interaction (Barr *et al*. 2013). We report standardized betas for all regressions serving as effect sizes. To interpret null findings, we computed Bayes Factors (BFs) of the relevant t-statistic (Rouder *et al*. 2009) using the BayesFactor R package (Morey and Rouder 2011). For analytic deviations from the preregistration, see online Supplementary Information G (p. 48).

### fMRI data acquisition

Anatomical and functional brain images were acquired with a 3 T Siemens TIM Trio scanner and a 12-channel phased-array head coil. T1-weighted MP-RAGE anatomical images were obtained for each subject (176 × 1 mm axial slices; field of view (FOV): 250 mm; repetition time (TR): 1900 ms; echo time (TE): 2520 ms; 256 × 256 matrix; 1 × 1 × 1 mm voxels). T2*-weighted functional images were collected using an echo-planar imaging sequence (46 x 3 mm transversal slices; TR: 2500 ms; TE: 30 ms; FOV: 192 mm; 64 × 64 matrix; 3 × 3 × 3 mm voxels).

### fMRI analysis

We selected three primary regions of interest (ROI) that encode affective and motivational features of experienced and empathic pain: the left AI (Fig. 2b), the right AI, and the dorsal ACC (dACC)/aMCC (Jackson *et al*. 2006a, 2006b, Ochsner *et al*. 2008, Fan *et al*. 2011, Lamm *et al*. 2011, Brethel-Haurwitz *et al*. 2018, O’Connell *et al*. 2019, Berluti *et al*. 2020). We used the same masks as O’Connell *et al*. 2019 (for preprocessing details and details on masks, see online Supplementary Information A).

For each participant, we applied a spherical searchlight (9-mm radius) using the RSA Toolbox in Python (Nili *et al*. 2014) to calculate pattern similarity maps across participants for the following conditions: self-pain (experienced pain > no experienced pain), other pain (observed pain > no observed pain), fearful anticipation of self-pain (experienced fearful anticipation of pain > no experienced anticipation of pain (safety trials)), and fearful anticipation of other pain (observed fearful anticipation of pain > no observed anticipation of pain (safety trials)) (Fig. 2b). Thus, trials in which pain was not delivered were subtracted from trials in which pain was delivered (i.e., pain − no-pain). Analyses were conducted using the three masks and in a whole-brain gray matter mask (online Supplementary Information A and E). Within each searchlight, we computed the Spearman’s rank correlation between the multi-voxel response patterns between (i) self and other pain and (ii) self and other fearful anticipation of pain. We then transformed these scores using the inverse hyperbolic tangent function (i.e., Fisher’s Z transformation) and assigned the Z-transformed similarity score to the center voxel. All searchlights required a minimum voxel inclusion threshold of 30%.

We used AFNI’s (Cox 1996) 3dBlurToFWHM to smooth univariate and pattern similarity maps to conduct two-sided one-sample *t*-tests within our regions of interests and across the whole brain. To assess pattern similarity across groups, AFNI’s 3dttest++ was employed with the -Clustsim flag to control for multiple comparisons (see online Supplementary Information A). To assess interactions between group and behavioral measures, we conducted ANOVAs (two-sided) using AFNI’s 3dMVM. In addition to small volume correction via family-wise error rate (FWER) we used AFNI’s 3dFWHMx function to estimate the spatial smoothness of population-level residuals obtained from 3dMVM. To interpret the strength of the evidence for null effects, we computed Bayes Factors (BF; see online Supplementary Information E). Finally, in parallel, we conducted similar analysis using a machine-learning based approach that employs multi-voxel cross-classification to decode self-other neural mapping (see online Supplementary Information F).

## Results

### Self-reported trait empathy is linked to loneliness and social connectedness at Time 1 and Time 2

Across the entire sample (*N* = 108), loneliness was significantly negatively correlated with trait empathy (PT), IOS, and well-being, whereas social connectedness was positively correlated with trait empathy (PT and EC), IOS, and well-being both pre- (Time 1; Table 1, see online supplementary material for a color version of this table) and post- (Time 2; Table 1) intervention. This is similar to prior findings (Beadle *et al*. 2012, Brethel-Haurwitz *et al*. 2018, O’Connell *et al*. 2019). Loneliness and social connectedness were correlated to such a high degree, *r*(106) = -0.77, *p* < .001, that we focused subsequent analyses on just loneliness [see online Supplementary Results, including Table 2B (see online supplementary material for a color version of this table) for additional results]. No differences in loneliness or empathy were found across LKM and PMR groups at Time 1 (see online Supplementary Information B, also for results regarding changes of IOS).

### Behavioral changes observed in self-reported trait loneliness but not empathy over time

**Figure 3:**
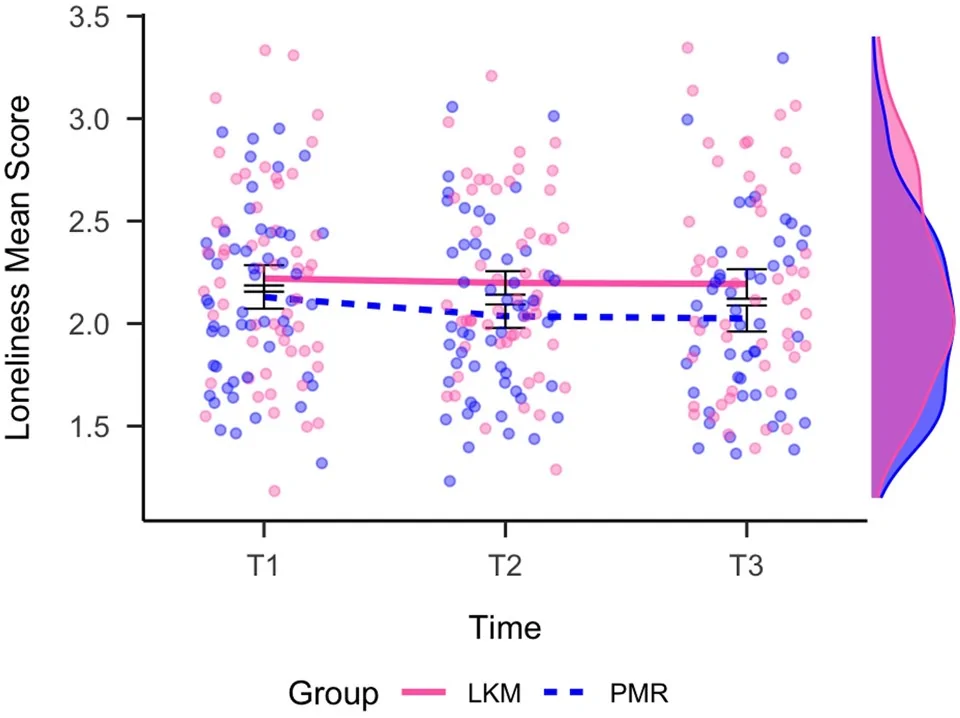
Loneliness changes over time with standard errors. *Note*. Across groups, there was a statistically small but significant main effect of time (Time 1 to Time 2) for loneliness that remained through Time 3. No credible evidence indicated changes due to intervention alone (the type of meditation training). The density plot displays the overall distribution of loneliness across time points. Pink (solid line) denotes LKM and blue (dashed line) denotes PMR (control) participants.

Longitudinal multilevel linear regression models conducted using the lme4 package (Bates *et al*. 2015) with random intercepts on the full sample (*N* = 108; 55 LKM, 53 PMR) (see online Supplementary Information B) assessed the main effects of time (Time 1 to Time 3), meditation intervention, and the time × intervention interaction, as well as demographic covariates (age, gender (female vs male, other), and education (college degree vs no college degree); Table 2, see online supplementary material for a color version of this table) on the dependent variables of interest. We found a main effect of time on loneliness (std. *β* = 0.14, SE = 0.07, *t*(201) = -2.06, 95% CI [−0.26, −0.01], *p* = .041), with loneliness decreasing from Time 1 to Time 2 and remaining stable thereafter (Fig. 3). We found no main effect of the intervention on loneliness across time points (std. *β*  =  0.31, SE = 0.17, *t*(102) = -1.86, 95% CI [−0.02, −0.64], *p* = .066; BF_01_=1.747; Table 2 and Table 7, see online supplementary material for a color version of this table). We also found no time × intervention interaction on loneliness (Time 1–Time 2: std. *β*  =  0.11, SE = 0.09, *t*(201) = 1.16, 95% CI [−0.07, 0.29], *p =* .247, BF_01_=6.565; Time 2–Time 3: std. *β*  =  0.06, SE = 0.09, *t*(201) = 0.62, 95% CI [−0.12, 0.23], *p* = .536; BF_01_=10.524; Table 7, see online supplementary material for a color version of this table) or changes in any trait empathy subscale over time (Tables 3 and 4, and Tables 8 and 9, see online supplementary material for a color version of these tables), which precluded us from running our preregistered mediation model investigating whether the effect of LKM on loneliness is driven by changes in empathy.

**Table 2:** Correlations between key variables at Time 2.

Variable	*M*	*SD*	1	2	3	4	5	6	7	8	9	10
1. SCS-R	4.45	0.77										
2. UCLA	2.12	0.43	−0.80***									
3. IOS	3.14	0.86	0.44***	−0.37***								
4. IRI EC	4.06	0.60	0.28**	−0.17	0.23*							
5. IRI F	3.55	0.81	−0.18	0.16	−0.08	0.22*						
6. IRI PD	2.41	0.72	−0.28**	0.19	−0.10	−0.05	0.16					
7. IRI PT	3.77	0.66	0.21*	−0.24*	0.28**	0.43***	0.07	0.00				
8. WB	7.71	1.23	0.48***	−0.51***	0.19*	0.12	−0.01	−0.14	0.19*			
9. Age	40.22	16.36	0.01	−0.06	−0.23*	0.05	−0.34***	−0.20*	−0.01	0.08		
10. Female	0.60	0.49	0.01	0.04	−0.06	0.17	0.16	0.22*	0.01	−0.13	−0.13	
11. Male	0.37	0.49	0.02	−0.08	0.07	−0.17	−0.16	−0.21*	0.00	0.13	0.12	−0.94***

*Note*. SCS-R, mean social connectedness; UCLA, mean loneliness; IOS, mean inclusion of other in the self; IRI EC, PD, PT, F, trait empathy measured using IRI with means of subscales empathic concern, personal distress, perspective taking, and fantasy; WB, mean well-being. Measures at Time 2, post-intervention.

* *P* < .05; ** *P* < .005; *** *P* < .001.

### Both LKM and PMR participants exhibit similar levels of neural empathy to a stranger’s pain

**Figure 4:**
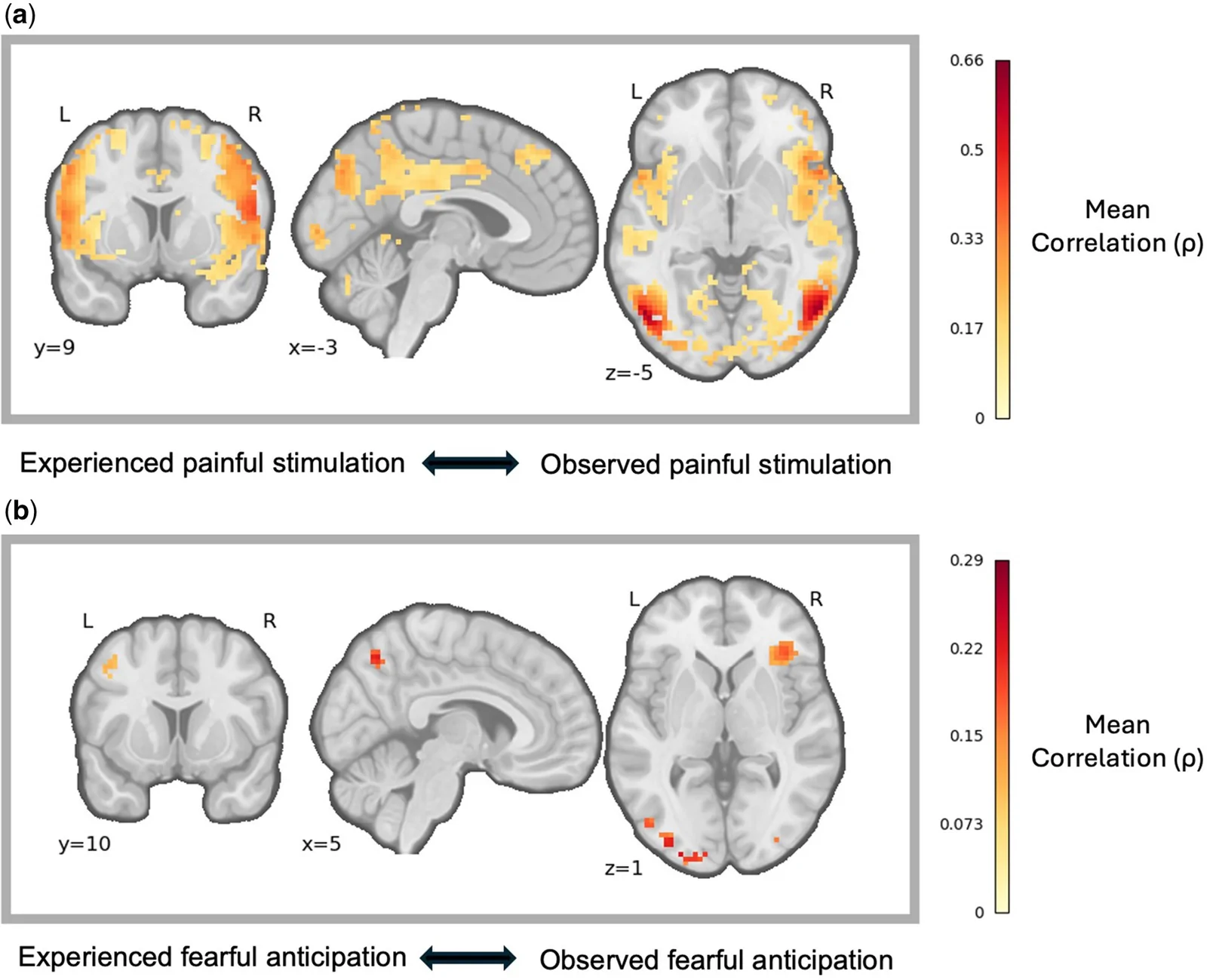
Whole-brain pattern similarity. *Note*. (a) Both LKM and PMR groups revealed significant pattern similarity between experienced and observed pain in regions including right AI/IFG, somatosensory cortex, motor cortex, superior parietal lobule, occipitotemporal cortex, and cerebellum. (b) Both LKM and PMR groups revealed pattern similarity between experienced and observed fearful anticipation of pain in regions including left visual cortex, left and right parietal-occipital sulcus, right AI, left premotor area, and right visual cortex (Table 39–41, see online supplementary material for a color version of these tables). Both *P* < .05_FWER_; underlying voxel height threshold *P* < .001.

Two-sided one-sample *t*-tests conducted in AFNI confirmed self-other pattern similarity in hypothesized regions of interest across the full sample (*N* = 54; 29 LKM, 25 PMR). Pattern similarity during experienced/observed pain was observed in left and right AI and dACC/aMCC, *p* < .001_FWER_ (Table 3). Pattern similarity was also observed during experienced/observed fearful anticipation in right AI, *p* < .001_FWER_ (Table 3). Whole brain analyses identified additional clusters in which pattern similarity was observed across groups, including right AI/IFG and regions of somatosensory, motor, and parietal cortex (Fig. 4) (Tables 36 and 38, see online supplementary material for a color version of these tables). But no credible evidence for effects of the intervention on empathic activation during either experienced/observed pain trials or experienced/observed fear trials was found in any of the ROIs, nor the whole brain, with moderate support for the null hypothesis (BF_01_ = 5.647–6.734; Tables 39 and 40, see online supplementary material for a color version of these tables).

**Table 3:** One-sample *t*-test shows statistically significant self-other pattern similarity in the AI and dACC.

			Pattern similarity (pain)							
*K*	Voxel Nr	Volume (mm^3^)	Peak x	Peak y	Peak z	*z*	*d*	Mean	SEM	Region
1	53	1431	0.5	−2.5	41.5	3.95	0.537	0.17	0.005	dACC
2	28	756	−5.5	−44.5	23.5	3.61	0.491	0.13	0.004	dACC
3	10	270	−5.5	−29.5	38.5	3.64	0.495	0.15	0.008	dACC
4	6	162	−2.5	−20.5	26.5	3.40	0.463	0.11	0.003	dACC
5	5	135	9.5	−50.5	11.5	3.58	0.487	0.12	0.007	dACC
6	5	135	6.5	−38.5	23.5	3.62	0.492	0.13	0.005	dACC
1	197	5319	45.5	0.5	2.5	4.17	0.568	0.15	0.002	Left AI
1	232	6264	−44.5	−23.5	−6.5	4.52	0.615	0.19	0.003	Right AI
			**Pattern similarity (fearful anticipation)**							
1	28	756	−35.5	−29.5	−0.5	3.85	0.524	0.12	0.004	Right AI

*Note.* Significant clusters self-other pattern similarity in the anterior insula (AI) and dorsal anterior cingulate cortex (dACC). *P* < .05_corr_; underlying voxel height threshold *P* < .001. *K* = cluster number.

### State empathy for fear/unpleasantness but not pain is linked to loneliness at Time 2

Pressure pain during pain trials was induced at *M* = 33.24 (SD = 14.25, range 10–65) pounds per square inch. After the task, self-reported pain was rated at *M* = 4.18 (SD = 1.19), fear at *M* = 3.28 (SD = 1.38), and unpleasantness at *M* = 3.69 (SD = 1.38). Participants rated their study partner’s pain at *M* = 4.06 (SD = 1.23), fear at *M* = 3.11 (SD = 1.45), and unpleasantness at *M* = 3.54 (SD = 1.41). No credible evidence for differences between LKM and PMR groups was found in ratings of pain, pain unpleasantness, and fear for either self or other (Table 10, see online supplementary material for a color version of this table).

Linear regressions with state empathy (i.e. similarity between one’s own and the study partner’s pain, fear, and unpleasantness, see online Supplementary Information A) and meditation group as independent predictors found that higher state empathy for fear as assessed in the post-scan survey outside of the scanner, was associated with less loneliness (std. *β* = −0.40, SE = 0.13, *t*(47) = −3.10, 95% CI [−0.65, −0.14], *p* = .003, Figure 2, see online supplementary material for a color version of this figure). We found no credible evidence that state empathy for fear was associated with self-reported trait empathy (EC or PT) or IOS for the study partner (BF_01_ 1.464 − 6.544 (Tables 11–18 and 33, see online supplementary material for a color version of these tables). State empathy for unpleasantness during painful stimulation was also associated with less loneliness (std. *β* = −0.28, SE = 0.13, *t*(47) = −2.13, 95% CI [−0.55, −0.02], *p* = .038), but not with other measures (BF_01_ = 4.437 − 6.724, Tables 19–25 and 34, see online supplementary material for a color version of these tables; though evidence for a relationship with social connectedness was anecdotal: BF_01_=1.346; Table 34, see online supplementary material for a color version of this table). State empathy for pain, which was assessed immediately after each run inside the scanner, was not associated with any measures (BF_01_ = 4.363 − 6.477; Tables 26–32 and 35, see online supplementary material for a color version of these tables).

### Neural empathy is linked to self-reported empathy but not loneliness at Time 2

We conducted an ANOVA using AFNI’s 3dMVM testing whether Time 2 loneliness or empathy (trait EC, PT, state empathy for fear, pain unpleasantness, or pain all at Time 2) correlated with multi-voxel pattern similarity within regions of interests across groups or differently for each group (Table 4). For each predictor, we ran a model testing the main effect of the mean-centered continuous predictor, the main effect of the categorical between-participants group variable (LKM vs PMR), and the interaction between the predictor and group with pattern similarity in each of the hypothesized region as the continuous outcome variable.

**Table 4:** ANOVA pattern similarity significant clusters.

Voxel Nr	Volume (mm^3^)	Peak x	Peak y	Peak z	*t*	*d*	SEM	Region
**Main effect empathic concern (EC) on pattern similarity (pain)**								
12	324	−2.5	−11.5	29.5	−3.99	0.543	0.122	dACC
**Main effect state empathy (pain rating) on pattern similarity (fearful anticipation)**								
8	216	30.5	−20.5	−12.5	3.73	0.508	0.064	Left AI
**Interaction effect state empathy (fear) × group on pattern similarity (fearful anticipation)**								
4	108	−41.5	−8.5	−6.5	3.63	0.494	0.042	Right AI
**Interaction state empathy (unpleasantness) × group on pattern similarity (fearful anticipation)**								
42	1134	−8.5	−44.5	8.5	3.88	0.528	0.043	dACC

*Note.* Significant clusters 3dMVM pattern similarity in the anterior insula (AI) and dorsal anterior cingulate cortex (dACC), as well as the whole brain for pain and fearful anticipation of pain. One cluster for each of the listed effects was found based on FWER (2 nearest neighbors (NN); *P* < .05_corr_ with underlying voxel height threshold *P* < .001).

**Figure 5:**
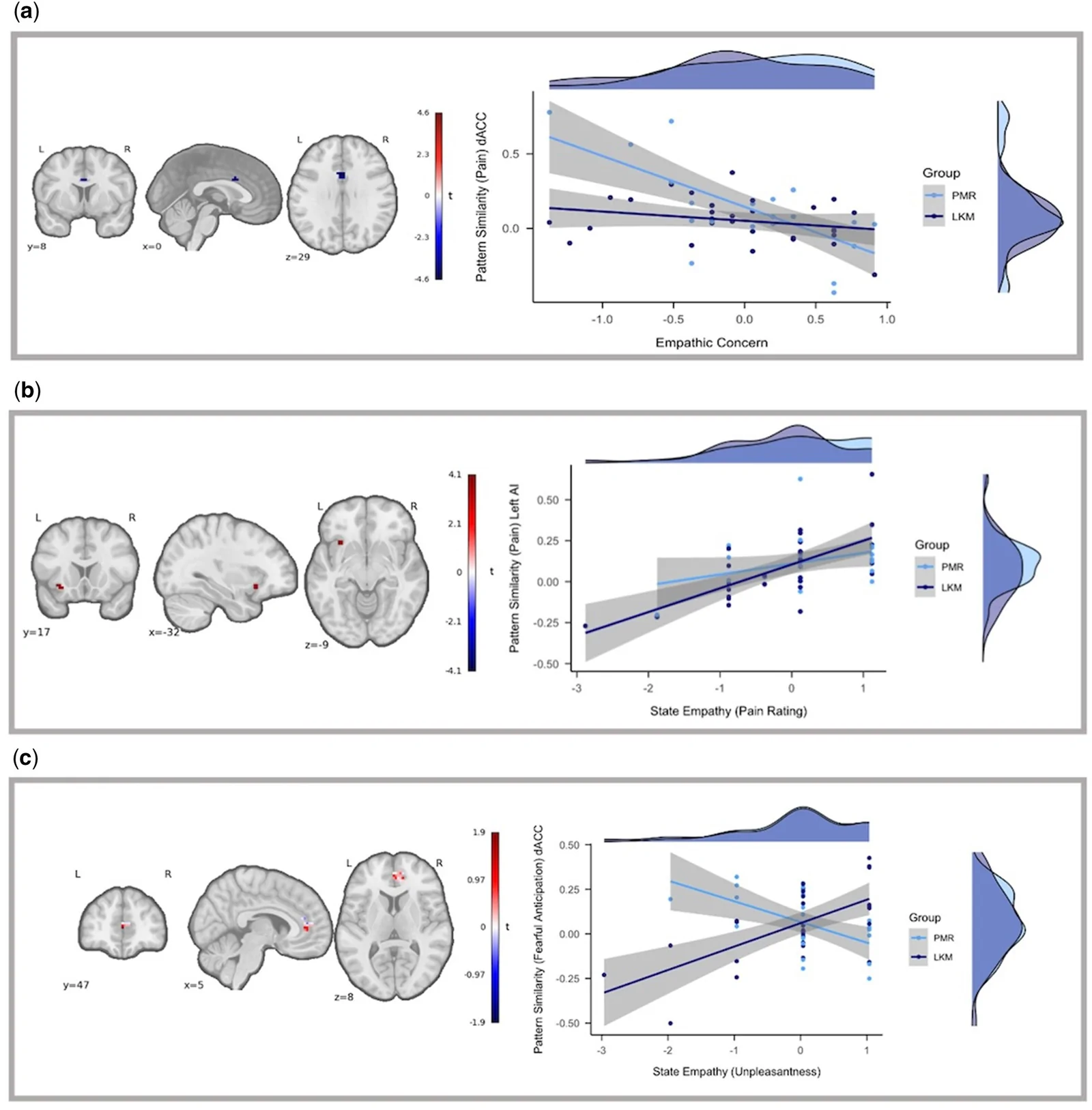
Pattern similarity interactions in AI and dACC. *Note*. (a) EC was negatively correlated with multi-voxel pattern similarity (pain) in the dACC (Table 4; volume 324 mm^3^). Visualization of the negative main effect of EC on pattern similarity (pain) in dACC. While the scatter plot seems to show this effect to be mainly driven by the active control (PMR), the interaction effect with the meditation group was not significant. Dots represent the subject-level mean similarity scores from the respective significant effect clusters. (b) State empathy (pain) was positively correlated with multi-voxel pattern similarity (pain) in the AI (Table 4; volume 216 mm^3^). Visualization of the main effect of state empathy (pain rating) on pattern similarity (pain) in left AI. The interaction effect was not significant. Dots represent the subject-level mean similarity scores from the respective significant effect clusters. (c) state empathy (unpleasantness) was correlated with pattern similarity (fearful anticipation) (Table 4; volume 1134 mm^3^). Visualization of the interaction effect of group and state empathy (fear) on pattern similarity (fearful anticipation) in the right AI. Individuals in the LKM group exhibited more pattern similarity during fearful anticipation, given that they also self-reported higher similarity of fearful anticipation of pain with the study partner. This effect was reversed for people in the PMR group. All scatter plots with error bars and data distribution were generated for visualization and interpretation only. Dots represent the subject-level mean similarity scores from the respective significant effect clusters extracted using 3dROIstats in AFNI.

We found a paradoxical main effect of trait empathy (EC) on pattern similarity in dACC during experienced/observed pain (*p *< 0.05_FWER_; underlying voxel height threshold *p *< 0.001) (Fig. 5a), such that individuals reporting *lower* EC showed *higher* pattern similarity during experienced/observed pain in dACC. This effect was more pronounced in participants who underwent PMR, although no statistically significant interaction was observed. We found no association between perspective taking (PT) and pattern similarity (BF_01_ = 4.968 − 6.546; Tables 42 and 43, see online supplementary material for a color version of these tables).

By contrast, we found a positive relationship between state empathy for pain and pattern similarity in left AI during experienced/observed pain (*p *< 0.05_FWER_; underlying voxel height threshold *p *< 0.001). Specifically, participants with higher state empathy (more similar self-other pain ratings) showed more pattern similarity during observed/experienced pain (Fig. 5b). We also found a group × state empathy (fear) interaction on pattern similarity (fearful anticipation) in right AI (Fig. 5c). Participants in the LKM group who reported higher state empathy for pain unpleasantness exhibited more pattern similarity during fearful anticipation, whereas this effect was reversed for the PMR group. We found no credible evidence for associations between loneliness and pattern similarity in left AI, right AI, or dACC, or any evidence for an interaction between group and loneliness being associated with pattern similarity (BF_01_ = 4.683 − 6.546 (Tables 41 and 42, see online supplementary material for a color version of these tables).

## Discussion

In a demographically representative sample of adults from the Washington, DC, region we found support for the previously identified association between greater loneliness and reduced self-reported trait empathy. During an empathic pain task, greater loneliness was also associated with reduced state empathy for a stranger’s fear of pain and pain unpleasantness but not with state empathy for pain delivery. We found little credible evidence that loneliness was associated with neural empathy, which we operationalized as multi-voxel pattern similarity during experienced and observed pain and fearful anticipation of pain. Although state empathy and neural empathy for pain were positively correlated, neither was correlated with self-reported trait empathy, which instead paradoxically correlated with *less* neural empathy for pain in dACC, a region that encodes affective and motivational features of experienced pain and empathy for others’ pain (Jackson *et al*. 2006a, 2006b, Ochsner *et al*. 2008, Fan *et al*. 2011, Lamm *et al*. 2011, Brethel-Haurwitz *et al*. 2018, O’Connell *et al*. 2019, Berluti *et al*. 2020). This effect was driven by participants in the PMR (control) group, although the interaction was not significant. We also found no credible support for the hypothesis that LKM training reduces loneliness through its effects on empathy. Participants randomized to both LKM and PMR training showed comparable reductions in loneliness but no changes in trait empathy through training. No credible evidence for specific effects of LKM on loneliness or on trait, state, or neural measures of empathy for pain was observed. Reductions in loneliness in both groups persisted throughout the 6-month Time 3 follow-up in the absence of further intervention.

Our results suggest that the relationship between loneliness and empathy is multifaceted. Similar to prior research, we found an inverse relationship between loneliness and self-reported empathy (Kalliopuska 1986, Beadle *et al*. 2012, Hu *et al*. 2020, Mwilambwe-Tshilobo *et al*. 2023). However, we did not observe consistent relationships between loneliness and state or neural measures of empathy. Reduced state empathy for fear and pain unpleasantness (assessed after the empathic pain task outside the scanner) was observed in lonelier adults. But loneliness was not associated with state empathy for pain assessed inside the scanner, nor with neural empathy for pain or fear. Together, these findings suggest that neural representations of others’ emotional experiences remain intact among lonelier people. But such people may perceive themselves to be less empathic. Consistent with this conclusion, prior work has found that empathic accuracy, emotion recognition, and other task-based measures of empathy are unimpaired in lonelier people (Di Tella *et al*. 2023, Mwilambwe-Tshilobo *et al*. 2023), who nonetheless subjectively rate their social skills as worse and may misperceive features of their social interactions (Wittenberg and Reis 1986). Given these accumulated findings, it may be that lonelier individuals’ difficulties are in part meta-cognitive, in that they underestimate their actual abilities to share and understand other people’s experiences and to connect with others.

Our results also highlight the value of considering multiple manifestations and measures of empathy, including trait, state, and neural empathy. The positive association we found between neural empathy for pain and state empathy for pain in left AI supports the conclusion these two measures are capturing a common phenomenon: the internal representation or simulation of another’s pain. By contrast, the *inverse* correlation we observed between neural empathy for pain in dACC and trait EC aligns with previous evidence that trait empathy, as measured by self-report, does not always correspond to other task-based measures of empathy (Murphy and Lilienfeld 2019). Indeed, we did not find trait EC or PT to be correlated with any other measure of state or neural empathy. This pattern of findings may in part reflect the fact that we operationalized state empathy in a way that paralleled our neural empathy measure: as self-other mapping in response to pain and fear induction. By contrast, the IRI (Davis 1983), like most trait empathy measures, assesses self-perceived tendencies to imagine and care about others’ internal states. While all of these operationalizations are captured under the broad accepted definition of empathy (Hall and Schwartz 2019), they likely capture partially distinct phenomena. State and trait empathy measures may also fail to correlate due to fundamental confounds in trait empathy scales, such as the fact that they ask participants how they would respond in imaginary empathy-eliciting situations and thus rely on visual imagery ability (Monzel *et al*. 2023). By contrast, state empathy is typically measured in response to physically present stimuli, as in our study, and so does not rely on imagery.

Moving forward, it may help to clarify our understanding of empathy and related phenomena to conceptualize self-report trait empathy measures as what they actually are: subjective reports of how empathic people believe themselves to be. In general, self-report measures are more accurate and predictive when people make intra-individual judgments (e.g., how much they enjoy reading versus socializing) than inter-individual phenomena (e.g., how much they enjoy socializing relative to other people) which requires accurate knowledge of not just the self, but of others (Corneille and Gawronski 2024). This conceptualization may help clarify why self-reported empathy so often fails to accurately predict inter-individual differences in behavior (Kanske *et al*. 2015, Brethel-Haurwitz *et al*. 2018). It may also help to inform interventions on empathy and loneliness. Lonely people may not require interventions to make them more empathic as much as interventions to increase their awareness of and confidence in their actual empathic capacities relative to other people. A meta-analysis indeed found the most effective loneliness interventions address maladaptive patterns of social cognition (Masi *et al*. 2011). Our findings add a specific maladaptive pattern of social cognition, rooted in less awareness in one’s own ability to empathize, to other recently found patterns like negative expectations about other people’s emotion transitions (Ma De Sousa *et al*. 2025) and feeling less empathized with by others (Pei *et al*. 2025). One possible mechanism to target in future research may be perceived similarity. Research shows that lonelier individuals neurally represent the world differently from their peers (Courtney and Meyer 2020, Baek *et al*. 2023). Self-reported empathy in turn is linked to perceived similarity between the target and perceiver (Batson 2005). Interventions targeting subjectively perceived similarity could perhaps increase self-reported empathy and decrease the feeling of being alone in the world.

Our results also suggest that features specific to an LKM intervention are unlikely to be effective in reducing loneliness by increasing empathy. We did not find LKM reduced loneliness relative to an active control condition. Indeed, the main effect of time on loneliness was primarily driven by PMR. This effect may reflect the fact that LKM also did not increase empathy as we hypothesized. The only effect of LKM on empathy we found was that completing LKM training increased the association between state empathy for fear and pattern similarity during fearful anticipation in the right AI. By contrast, both LKM and PMR groups exhibited a positive association between state empathy for pain and neural empathy for pain in left AI. These findings support the validity of our task-based measures of state empathy, but not that empathy is notably increased by LKM training. One possible interpretation of the observed interaction between state empathy and neural empathy for fear following LKM is that participants in this condition became more subjectively attuned to others’ fear, causing more alignment between neural and state empathy. However, this exploratory finding would require further testing.

It will also require further testing to determine why both LKM and PMR interventions caused reductions in loneliness. One possibility is that both interventions improved one or more unmeasured variables, such as stress, anxiety, or depression (Muhammad Khir *et al*. 2024). It is also plausible that repeated positive interactions with researchers throughout the multi-week research study contributed to reduced loneliness. In addition, participants may have derived psychological benefits, such as a sense of pride or empowerment, from participating, as previous work indicates is common among research participants (Castillo *et al*. 2012, Lakeman *et al*. 2013). If true, this would also help explain why a recent meta-analysis found that studies of contemplative practices that include an active vs passive waitlist control see less benefit on compassion (Kreplin *et al*. 2018). Finally, our results could reflect a placebo effect driven by participants’ expectations of benefit, increased attention to their emotional states, or the belief that they were engaging in a meaningful self-improvement activity, rather than by the specific mechanisms of either intervention. Without an additional passive waitlist control group, the answer cannot be determined with certainty.

One possible reason why the LKM intervention did not increase empathy could be that variability in empathic behavior may depend less on ability and more on motivation (Zaki 2014, Cameron 2018), for example, whether individuals care about the target. In general, people naturally empathize more with targets they care more about (Hein *et al*. 2010). From this perspective, interventions that aim to increase empathy per se may be less effective than those that increase social motivation, perceived similarity, or caring. Although LKM was often proposed as one such intervention, neither the present study nor our prior work provides strong evidence that it produces durable increases in empathy.

Some additional limitations should be considered when interpreting the results of this study. First, trait empathy was assessed both before and after the interventions, but state empathy and neural empathy only after the intervention. This prevents us from calculating pre-post changes in these outcomes or linking these to pre-post changes in other variables. Thus, we cannot determine whether, for example, baseline associations between loneliness and neural empathy were present but attenuated by training. Although scanning participants twice is resource intensive, future studies could include baseline neural measures to assess whether associations between loneliness and neural empathy are present prior to intervention and to track how these relationships change over time. It is also possible that our task failed to capture individual variance related to empathy for positive emotions, as it focused exclusively on empathy for negative emotions (e.g., pain and fear). Some evidence suggests that loneliness may be more strongly related to biases in processing positive emotional information (Igarashi 2025), suggesting that future research may want to investigate the relationship between loneliness and neural empathy for positive emotions. Additionally, the meditation training was carried out online, which is an accessible, scalable format. However, variables related to how participants complete online meditation training (presence of distractors or other simultaneous activities) could not be assessed in our study. Future research should explore how factors such as motivation and the delivery of meditation practices may affect the effectiveness of these interventions. Lastly, it is important to distinguish between the statistical and clinical significance of our findings. The small effect sizes associated with changes in loneliness that we observed may not represent clinically significant changes, underscoring the need for future work to examine whether these changes translate into meaningful real-world or long-term outcomes.

## Conclusion

In this pre-registered RCT, we assessed whether LKM training can reduce loneliness by increasing empathy. We found no evidence that LKM promotes empathy or reduces loneliness compared to an active control—although loneliness declined across participants in both conditions. Greater loneliness was associated with reduced trait empathy measured via self-report, as well as with reduced state empathy for fear and pain unpleasantness but was not associated with state empathy for pain or any measure of neural empathy for pain or fearful anticipation of pain. Thus, our findings do not support the efficacy of LKM interventions in reducing loneliness through empathy but instead support the possibility that the recognition and reduction of subjective empathic deficits in loneliness could improve long-term outcomes and quality of life by enabling the development of targeted subjective empathy interventions.

## Supplementary Material

nsag015_Supplementary_Data

## Data Availability

Access to data, materials, analysis code, and the preregistration can be found in the study’s online repository, linked to GitHub (https://osf.io/2sx3v). fMRI data in BIDS format are available at https://openneuro.org/datasets/ds006243. Analyses were conducted in Python, bash, R/RStudio, and MATLAB.
