# Supplementary material for: Lonelier people feel less empathic despite intact neural empathy responses after meditation training
Source: Soc Cogn Affect Neurosci. 2026 Jun 20;21(1):nsag015. doi: 10.1093/scan/nsag015 (PMC13282970; doi:10.1093/scan/nsag015)
Supplement: nsag015_Supplementary_Data [file nsag015_supplementary_data.doc]

# **Supplemental Information:**

**Lonelier people feel less empathic despite intact neural empathy responses after meditation training**

## **Supplemental Information A: Detailed Methods**

We assessed state empathy by calculating the absolute value of the other-self difference scores from participants’ ratings of pain during, and retrospective ratings of fear, and unpleasantness of, aversive trials, which were subsequently multiplied times -1 such that a higher score would mean more similarity (Figure 1). All raw ratings were on a 7-point Likert scale (1 = "not at all,” 7 = "extremely painful/fearful/unpleasant).

**Figure 1**

*Overview of Empathy Measures*


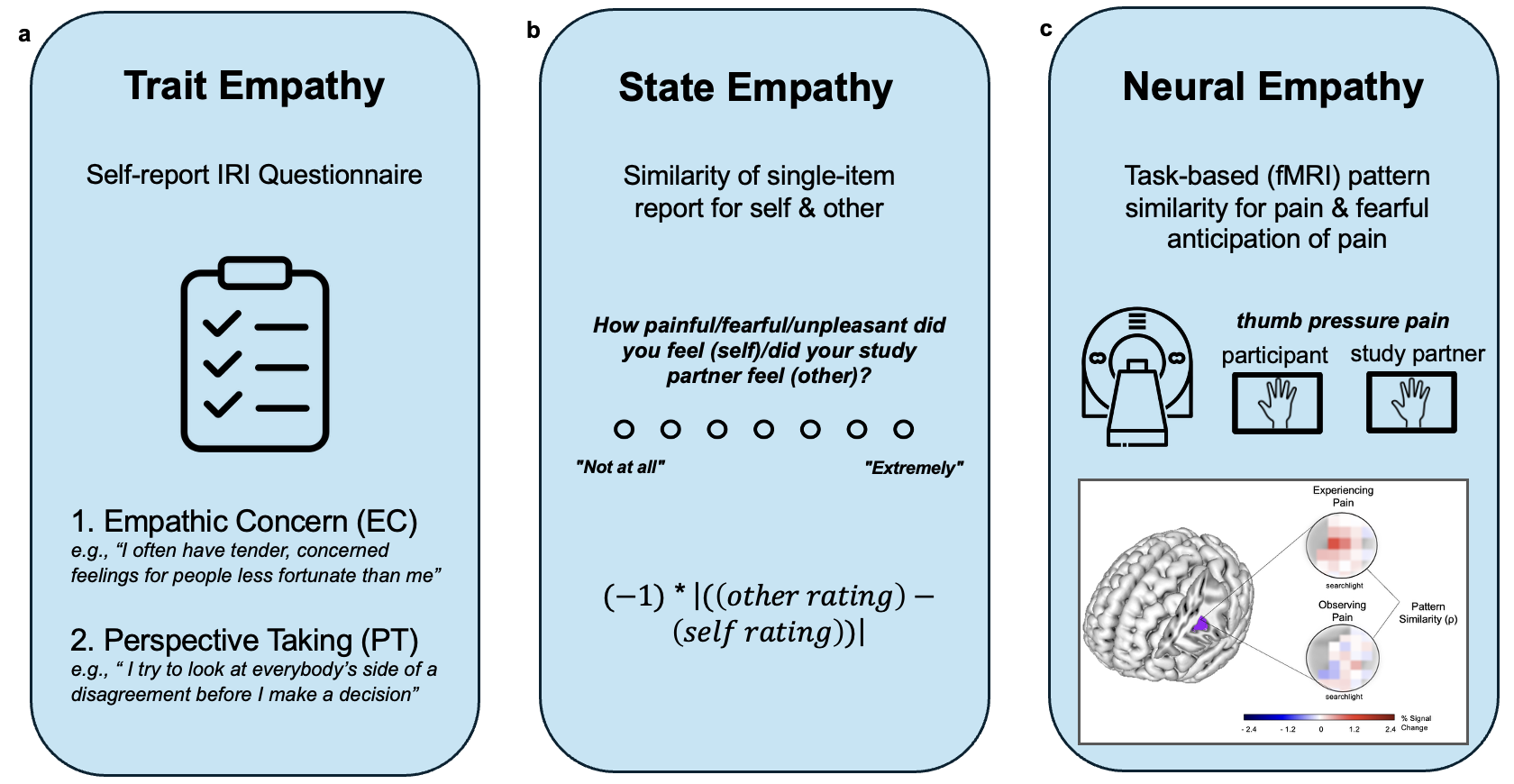


*Note*. At each time point (T1-T3), **a)** Trait Empathy via the Interpersonal Reactivity Index (IRI; Davis, 1983) was measured. Just after the intervention, half of participants underwent fMRI scanning, during which we measured **b)** neural empathic responding (multi-voxel pattern similarity) using the empathic pain task. We also measured **c)** state empathy for pain, which was reported verbally and based on the similarity of the participants’ rating of their own and their study partner’s pain after each run in the scanner. State empathy for fear and unpleasantness was asked after the scan in a survey and was based on the similarity of the participant’s fear rating during anticipation and pain unpleasantness during painful stimulation for themselves and their study partner respectively. Our behavioral measure was designed to mirror this principle as closely as possible: smaller discrepancies between experienced and observed pain indicate more simulation and, consequently, more affective alignment between self and other, which we conceptualize as higher state empathy. By incorporating the self-rating, the difference score more directly captures how much the participant’s own state anchors their representation of the other’s state.

## **Exclusions**

Out of the 183 participants recruited, 75 were excluded from the full sample for reasons including completing less than 75% of the meditations (n = 29; overall percentage of meditation completed in the final sample was M = 94.36%, SD = 6.58%), substance use (n = 11), failing more than two attention checks in the screener survey (n = 5), having prior experience with LKM (n = 1), and other reasons (n = 9), including ineligibility for fMRI scanning, non-responsiveness to follow-up communications about the meditations or voluntary withdrawal from the study. To ensure the experimental groups were representative of the Washington DC Metropolitan area and demographically matched, an additional 20 participants were excluded from the full sample.

Eight of these participants were later excluded, due to falling asleep during scanning (n = 1), technical issues during scanning (n = 1), not completing meditations as instructed (n = 1), fMRI contraindications on scanning day (n = 1), knowing the confederate (n = 1), and incomplete scanning due to anxiety or claustrophobia (n = 3). All participants were below our threshold for excessive motion during the MRI scan (>15% TRs with >0.5 mm head-movement). To preserve statistical power for the fMRI analysis, we did not exclude fMRI participants from any analysis, who failed attention checks during the survey.

Of the 108 participants who completed the intervention and the post-intervention assessment (T2), 97 also completed the 6-month follow-up survey (T3), with 11 lost to follow-up. Missing T3 data were handled via maximum likelihood estimation in the linear mixed-effects models.

**Empathic Pain Task**

After entering the scanner, thumbnail pressure-pain stimulation was adjusted to a subjective threshold of “slightly intense” pain for each participant (a rating of 13.5 on a 21-point Gracely Box Scale; Gracely et al., 1992) with a safety limit set at a maximum of 80 pounds per square inch (PSI). The stimulation was applied using a 1.9-cm² plastic rubber probe to the lunula (the whitish, crescent-shaped region) at the base of the participant's right thumbnail. A pneumatic device in the console room outside the scanner (connected via a flexible tube) provided thumbnail pressure to the participant in the scanner with an accuracy of 0.1 PSI.

Auditory and pressure pain stimuli were presented using E-Prime 2.0 (Schneider et al., 2002). Audio cues (beeps or buzzes depending on the type of mapping) were presented in a random order to indicate trial conditions. In one mapping, a single beep signaled a fear trial (potential upcoming painful stimulation), and two beeps indicated a safety trial (no upcoming painful stimulation), while one buzz signaled a pain trial (painful stimulation currently occurring) and two buzzes indicated no pain (no current stimulation). In the alternate mapping, these meanings were reversed such that a single buzz signaled a fear trial (potential upcoming painful stimulation), and two buzzes indicated a safety trial (no upcoming painful stimulation), while one beep signaled a pain trial (painful stimulation currently occurring) and two beeps indicated no pain (no current stimulation). Participants learned the meaning of the audio cues prior to the task. The study partner heard the same audio cues as the participant and was fully aware of the task paradigm. The study partner received painful stimulation to their hand using the same procedure and device while sitting in the console room outside of the scanner. However, the PSI for the study partner’s thumbnail pressure was standardized at 15 PSI, which was within the range of levels selected by participants. The specific task version was also randomized (2 possible randomization versions).

**fMRI Preprocessing & Analysis**

fMRI data were converted into BIDS format (Gorgolewski et al., 2016), and the functional images were then preprocessed using fMRIPrep version 23.0.0 (Esteban et al., 2019). This included anatomical T1-weighted brain extraction, anatomical surface extraction, head-motion estimation and correction, susceptibility-derived distortion estimation and unwarping, slice-timing correction, intrasubject registration, and spatial normalization (intersubject registration). The first four volumes at the start of each run were discarded. Each run contained 15 fearful anticipation events, 15 non-fearful anticipation events, and, due to the probabilistic nature of the pain stimulus, 10 pain events and 20 no-pain events. AFNI’s (Cox, 1996) 3ddeconvolve function was used to fit a General Linear Model to the unsmoothed time series and generate parameter estimates for the experienced pain, no experienced pain, experienced fearful anticipation, no experienced fearful anticipation, observed pain, no observed pain, observed fearful anticipation and no observed fearful anticipation events.

For ROIs, we used the left anterior insula (AI) (Figure 3a), right anterior insula, and dorsal anterior cingulate cortex/anterior middle cingulate cortex (dACC/aMCC), for which we used the same masks as O’Connell et al. (2019), which were modified from the automated anatomical labeling atlas (AAL; Tzourio-Mazoyer et al., 2002) where right and left masks included insular labeled regions, and were constrained to be anterior to y = 67. For the dACC/aMCC mask, both the anterior cingulate and the middle cingulate cortex labeled regions were included and this mask was also constrained to be anterior to y = 0 based on Neurosynth reverse inference maps for “fear” and “pain” (Vogt, 2016; Yarkoni et al., 2011).

To assess pattern similarity across groups, AFNI's 3dttest++ was employed with the -Clustsim flag to control for multiple comparisons. This option conducts nonparametric permutation testing with 10,000 Monte Carlo simulations to determine cluster-level significance thresholds. Significant clusters were identified using an initial voxelwise threshold of p < .001, followed by cluster-size thresholding that controlled the family-wise error rate (FWER) at p < .05 with AFNI's 3dClusterize. This approach was developed to reduce the false positive rate in response to Eklund et al. (2016).

## **Supplemental Information B: Behavioral Changes in Self-reported Trait Loneliness but Not Empathy**

**Table 1**

*Correlations Between Key Variables at Time 2*

| *Variable* | *M* | *SD* | *1* | *2* | *3* | *4* | *5* | *6* | *7* | *8* | *9* | *10* |
| --- | --- | --- | --- | --- | --- | --- | --- | --- | --- | --- | --- | --- |
| 1. SCS-R | 4.36 | 0.83 |  |  |  |  |  |  |  |  |  |  |
| 2. UCLA | 2.18 | 0.45 | -0.77*** |  |  |  |  |  |  |  |  |  |
| 3. IOS | 2.93 | 0.84 | 0.31** | -0.28** |  |  |  |  |  |  |  |  |
| 4. IRI EC | 4.01 | 0.58 | 0.24* | -0.13 | 0.24* |  |  |  |  |  |  |  |
| 5. IRI F | 3.49 | 0.85 | -0.19* | 0.21* | -0.22* | 0.31** |  |  |  |  |  |  |
| 6. IRI PD | 2.49 | 0.76 | -0.25* | 0.29** | -0.17 | 0.07 | 0.23* |  |  |  |  |  |
| 7. IRI PT | 3.66 | 0.75 | 0.25** | -0.24* | 0.18 | 0.41*** | 0.01 | -0.09 |  |  |  |  |
| 8. WB | 7.33 | 1.31 | 0.59*** | -0.57*** | 0.30** | 0.19 | -0.06 | -0.29** | 0.13 |  |  |  |
| 9. Age | 40.22 | 16.36 | 0.11 | -0.20* | -0.10 | -0.01 | -0.37*** | -0.28** | -0.03 | 0.03 |  |  |
| 10. Female | 0.6 | 0.49 | 0.00 | 0.03 | -0.02 | 0.15 | 0.17 | 0.16 | -0.07 | -0.02 | -0.13 |  |
| 11. Male | 0.37 | 0.49 | 0.04 | -0.07 | 0.03 | -0.14 | -0.19* | -0.13 | 0.05 | 0.03 | 0.12 | -0.94*** |

Note: *p < .05; **p < .005; ***p < .001. Social Connectedness (SCS-R),

Loneliness (UCLA), Mean Inclusion of Other in the Self (IOS), Trait Empathy measured using IRI with subscales Empathic Concern (EC), Personal Distress (PD), Perspective Taking (PT), Fantasy (F), Mean Well-being (WB).

### Frequentist Mixed Models Group Differences Over Time

| **Table 2**  *Loneliness ~ time * GroupID + age + Male + Other + YesCollegeDegree*  Fixed Effects |
| --- |

| *Predictors* | *b* | *SE* | *std. b* | *std. SE* | *95% CI* | *t* | *p* | *df* |
| --- | --- | --- | --- | --- | --- | --- | --- | --- |
| (Intercept) | 2.17 | 0.12 | -0.15 | 0.12 | 1.94 – 2.39 | 18.82 | **<0.001** | 201 |
| time [1] | -0.06 | 0.03 | -0.14 | 0.07 | -0.12 – -0.00 | -2.06 | **0.041** | 201 |
| time [2] | -0.03 | 0.03 | -0.06 | 0.06 | -0.09 – 0.03 | -1.01 | 0.314 | 201 |
| GroupID [0.5] | 0.14 | 0.08 | 0.31 | 0.17 | -0.01 – 0.29 | 1.86 | 0.066 | 102 |
| age | -0.00 | 0.00 | -0.14 | 0.08 | -0.01 – 0.00 | -1.70 | 0.092 | 102 |
| Male | -0.07 | 0.08 | -0.08 | 0.08 | -0.22 – 0.08 | -0.90 | 0.368 | 102 |
| Other | 0.31 | 0.23 | 0.12 | 0.08 | -0.13 – 0.75 | 1.38 | 0.170 | 102 |
| YesCollegeDegree | 0.10 | 0.09 | 0.09 | 0.08 | -0.08 – 0.28 | 1.10 | 0.275 | 102 |
| time [1] × GroupID [0.5] | 0.05 | 0.04 | 0.11 | 0.09 | -0.03 – 0.13 | 1.16 | 0.247 | 201 |
| time [2] × GroupID [0.5] | 0.02 | 0.04 | 0.06 | 0.09 | -0.05 – 0.10 | 0.62 | 0.536 | 201 |

| Random Effects | |
| --- | --- |
| σ^2^ | 0.02 |
| τ_00_ _SubID_ | 0.14 |
| τ_11_ _SubID.time1_ | 0.03 |
| τ_11_ _SubID.time2_ | 0.03 |
| ρ_01_ | 0.07 |
|  | 0.23 |
| ICC | 0.90 |
| N _SubID_ | 108 |
| Observations | 313 |
| Marginal R^2^ / Conditional R^2^ | 0.070 / 0.910 |
| AIC | 255.180 |

*Note*. This random intercept model shows the change in loneliness (UCLA; Russell, 1996) from T1 to T2 and T2 to T3. Time is a factor with three levels (T1, T2, T3). GroupID is centered (LKM = 0.5, PMR = -0.5). Demographics are dummy-coded (gender reference group female, education reference group no college degree). We report standardized betas for all regressions. N = 108 at T1 and T2; n = 97 at T3. Missing T3 data were handled via maximum likelihood estimation in lme4. Total observations = 313.

| **Table 2B**  *Social Connectedness ~ time * GroupID + age + Male + Other + YesCollegeDegree*  Fixed Effects |
| --- |

| *Predictors* | *b* | *SE* | *std. b* | *std. SE* | *95% CI* | *t* | *p* | *df* |
| --- | --- | --- | --- | --- | --- | --- | --- | --- |
| (Intercept) | 4.54 | 0.22 | 0.15 | 0.13 | 4.11 – 4.97 | 20.63 | **<0.001** | 201 |
| time [1] | 0.09 | 0.05 | 0.11 | 0.06 | -0.00 – 0.18 | 1.85 | 0.065 | 201 |
| time [2] | 0.06 | 0.05 | 0.07 | 0.06 | -0.04 – 0.15 | 1.18 | 0.241 | 201 |
| GroupID [0.5] | -0.25 | 0.14 | -0.31 | 0.18 | -0.53 – 0.03 | -1.75 | 0.084 | 102 |
| age | 0.00 | 0.00 | 0.07 | 0.09 | -0.01 – 0.01 | 0.78 | 0.438 | 102 |
| Male | 0.05 | 0.15 | 0.03 | 0.09 | -0.25 – 0.34 | 0.32 | 0.753 | 102 |
| Other | -0.40 | 0.43 | -0.08 | 0.09 | -1.25 – 0.45 | -0.92 | 0.360 | 102 |
| YesCollegeDegree | -0.19 | 0.17 | -0.10 | 0.09 | -0.54 – 0.15 | -1.12 | 0.267 | 102 |
| time [1] × GroupID [0.5] | -0.07 | 0.07 | -0.09 | 0.08 | -0.20 – 0.05 | -1.12 | 0.265 | 201 |
| time [2] × GroupID [0.5] | -0.09 | 0.07 | -0.12 | 0.08 | -0.22 – 0.04 | -1.40 | 0.164 | 201 |

| Random Effects | |
| --- | --- |
| σ^2^ | 0.05 |
| τ_00_ _SubID_ | 0.51 |
| τ_11_ _SubID.time1_ | 0.08 |
| τ_11_ _SubID.time2_ | 0.07 |
| ρ_01_ | -0.04 |
|  | 0.04 |
| ICC | 0.92 |
| N _SubID_ | 108 |
| Observations | 313 |
| Marginal R^2^ / Conditional R^2^ | 0.047 / 0.923 |
| AIC | 564.808 |
| *Note*. This random intercept model shows the change in loneliness (SCS-R; Lee et al., 2001) from T1 to T2 and T2 to T3. Time is a factor with three levels (T1, T2, T3). GroupID is centered (LKM = 0.5, PMR = -0.5). Demographics are dummy-coded (gender reference group female, education reference group no college degree). We report standardized betas for all regressions. | |

| **Table 3**  *Trait Empathic Concern (IRI) ~ time * GroupID + age + Male + Other + YesCollegeDegree* |
| --- |

Fixed Effects

| *Predictors* | *b* | *SE* | *std. b* | *std. SE* | *95% CI* | *t* | *p* | *df* |
| --- | --- | --- | --- | --- | --- | --- | --- | --- |
| (Intercept) | 4.06 | 0.16 | 0.04 | 0.13 | 3.74 – 4.37 | 24.83 | **<0.001** | 201 |
| time [1] | -0.00 | 0.04 | -0.01 | 0.06 | -0.07 – 0.06 | -0.12 | 0.901 | 201 |
| time [2] | -0.04 | 0.04 | -0.06 | 0.06 | -0.11 – 0.03 | -1.00 | 0.319 | 201 |
| GroupID [0.5] | -0.03 | 0.11 | -0.05 | 0.18 | -0.24 – 0.18 | -0.29 | 0.774 | 102 |
| age | 0.00 | 0.00 | 0.04 | 0.09 | -0.01 – 0.01 | 0.40 | 0.691 | 102 |
| Male | -0.21 | 0.11 | -0.16 | 0.09 | -0.42 – 0.01 | -1.86 | 0.065 | 102 |
| Other | -0.17 | 0.32 | -0.05 | 0.09 | -0.80 – 0.46 | -0.54 | 0.593 | 102 |
| YesCollegeDegree | 0.02 | 0.13 | 0.01 | 0.09 | -0.23 – 0.27 | 0.16 | 0.875 | 102 |
| time [1] × GroupID [0.5] | 0.05 | 0.05 | 0.09 | 0.08 | -0.04 – 0.15 | 1.10 | 0.273 | 201 |
| time [2] × GroupID [0.5] | 0.06 | 0.05 | 0.10 | 0.08 | -0.04 – 0.16 | 1.18 | 0.241 | 201 |

| Random Effects | |
| --- | --- |
| σ^2^ | 0.03 |
| τ_00_ _SubID_ | 0.28 |
| τ_11_ _SubID.time1_ | 0.04 |
| τ_11_ _SubID.time2_ | 0.04 |
| ρ_01_ | 0.15 |
|  | 0.18 |
| ICC | 0.91 |
| N _SubID_ | 108 |
| Observations | 313 |
| Marginal R^2^ / Conditional R^2^ | 0.032 / 0.916 |
| AIC | 400.258 |

*Note*. This random intercept model shows the change in empathic concern (IRI; Davis, 1983) from T1 to T2 and T2 to T3. Time is a factor with three levels (T1, T2, T3). GroupID is centered (LKM = 0.5, PMR = -0.5). Demographics are dummy-coded (gender reference group female, education reference group no college degree). We report standardized betas for all regressions.

| **Table 4**  *Trait Perspective Taking (IRI) ~ time * GroupID + age + Male + Other + YesCollegeDegree* |
| --- |

Fixed Effects

| *Predictors* | *b* | *SE* | *std. b* | *std. SE* | *95% CI* | *t* | *p* | *df* |
| --- | --- | --- | --- | --- | --- | --- | --- | --- |
| (Intercept) | 3.94 | 0.19 | -0.00 | 0.13 | 3.57 – 4.30 | 21.01 | **<0.001** | 201 |
| time [1] | 0.05 | 0.05 | 0.07 | 0.07 | -0.04 – 0.14 | 1.04 | 0.302 | 201 |
| time [2] | 0.01 | 0.04 | 0.01 | 0.06 | -0.08 – 0.09 | 0.13 | 0.896 | 201 |
| GroupID [0.5] | -0.00 | 0.12 | -0.00 | 0.18 | -0.24 – 0.24 | -0.02 | 0.986 | 102 |
| age | -0.00 | 0.00 | -0.07 | 0.09 | -0.01 – 0.00 | -0.81 | 0.421 | 102 |
| Male | 0.02 | 0.13 | 0.01 | 0.09 | -0.23 – 0.27 | 0.16 | 0.872 | 102 |
| Other | 0.02 | 0.37 | 0.01 | 0.09 | -0.69 – 0.74 | 0.06 | 0.950 | 102 |
| YesCollegeDegree | -0.12 | 0.15 | -0.07 | 0.09 | -0.41 – 0.17 | -0.82 | 0.415 | 102 |
| time [1] × GroupID [0.5] | 0.04 | 0.06 | 0.06 | 0.09 | -0.08 – 0.17 | 0.64 | 0.522 | 201 |
| time [2] × GroupID [0.5] | 0.04 | 0.06 | 0.06 | 0.09 | -0.08 – 0.16 | 0.68 | 0.500 | 201 |

| Random Effects | |
| --- | --- |
| σ^2^ | 0.04 |
| τ_00_ _SubID_ | 0.37 |
| τ_11_ _SubID.time1_ | 0.08 |
| τ_11_ _SubID.time2_ | 0.06 |
| ρ_01_ | -0.18 |
|  | -0.11 |
| ICC | 0.91 |
| N _SubID_ | 108 |
| Observations | 313 |
| Marginal R^2^ / Conditional R^2^ | 0.018 / 0.912 |
| AIC | 510.067 |

*Note*. This random intercept model shows the change in perspective taking (IRI; Davis, 1983) from T1 to T2 and T2 to T3. Time is a factor with three levels (T1, T2, T3). GroupID is centered (LKM = 0.5, PMR = -0.5). Demographics are dummy-coded (gender reference group female, education reference group no college degree). We report standardized betas for all regressions.

| **Table 5**  *Mean IOS ~ time * GroupID + age + Male + Other + YesCollegeDegree* |
| --- |

Fixed Effects

| *Predictors* | *b* | *SE* | *std. b* | *std. SE* | *95% CI* | *t* | *p* | *df* |
| --- | --- | --- | --- | --- | --- | --- | --- | --- |
| (Intercept) | 3.61 | 0.21 | 0.06 | 0.12 | 3.19 – 4.02 | 16.91 | **<0.001** | 201 |
| time [1] | 0.13 | 0.06 | 0.15 | 0.07 | 0.02 – 0.25 | 2.21 | **0.028** | 201 |
| time [2] | 0.04 | 0.06 | 0.04 | 0.07 | -0.07 – 0.15 | 0.66 | 0.509 | 201 |
| GroupID [0.5] | -0.08 | 0.14 | -0.10 | 0.16 | -0.35 – 0.19 | -0.59 | 0.558 | 102 |
| age | -0.00 | 0.00 | -0.03 | 0.08 | -0.01 – 0.01 | -0.40 | 0.693 | 102 |
| Male | 0.18 | 0.15 | 0.10 | 0.08 | -0.10 – 0.47 | 1.26 | 0.211 | 102 |
| Other | -0.31 | 0.42 | -0.06 | 0.08 | -1.13 – 0.51 | -0.74 | 0.460 | 102 |
| YesCollegeDegree | -0.62 | 0.17 | -0.31 | 0.08 | -0.95 – -0.29 | -3.68 | **<0.001** | 102 |
| time [1] × GroupID [0.5] | 0.04 | 0.08 | 0.05 | 0.10 | -0.12 – 0.20 | 0.47 | 0.636 | 201 |
| time [2] × GroupID [0.5] | 0.11 | 0.08 | 0.13 | 0.09 | -0.04 – 0.27 | 1.44 | 0.152 | 201 |

| Random Effects | |
| --- | --- |
| σ^2^ | 0.06 |
| τ_00_ _SubID_ | 0.47 |
| τ_11_ _SubID.time1_ | 0.14 |
| τ_11_ _SubID.time2_ | 0.10 |
| ρ_01_ | 0.08 |
|  | 0.02 |
| ICC | 0.90 |
| N _SubID_ | 108 |
| Observations | 313 |
| Marginal R^2^ / Conditional R^2^ | 0.125 / 0.913 |
| AIC | 654.704 |

*Note*. This random intercept model shows the change in average inclusion of other in the self (IOS; Aron et al., 1992) from T1 to T2 and T2 to T3. Time is a factor with three levels (T1, T2, T3). GroupID is centered (LKM = 0.5, PMR = -0.5). Demographics are dummy-coded (gender reference group female, education reference group no college degree). We report standardized betas for all regressions.

| **Table 6**  *IOS (Stranger) ~ time * GroupID + age + Male + Other + YesCollegeDegree* |
| --- |

Fixed Effects

| *Predictors* | *b* | *SE* | *std. b* | *std. SE* | *95% CI* | *t* | *p* | *df* |
| --- | --- | --- | --- | --- | --- | --- | --- | --- |
| (Intercept) | 1.89 | 0.17 | -0.00 | 0.10 | 1.56 – 2.22 | 11.24 | **<0.001** | 201 |
| time [1] | 0.16 | 0.07 | 0.21 | 0.09 | 0.02 – 0.30 | 2.29 | **0.023** | 201 |
| time [2] | 0.04 | 0.06 | 0.05 | 0.07 | -0.07 – 0.14 | 0.67 | 0.506 | 201 |
| GroupID [0.5] | 0.01 | 0.11 | 0.01 | 0.15 | -0.20 – 0.22 | 0.09 | 0.928 | 102 |
| age | -0.01 | 0.00 | -0.18 | 0.07 | -0.01 – -0.00 | -2.36 | **0.020** | 102 |
| Male | 0.23 | 0.11 | 0.15 | 0.07 | 0.01 – 0.45 | 2.03 | **0.045** | 102 |
| Other | -0.34 | 0.33 | -0.08 | 0.07 | -0.98 – 0.30 | -1.03 | 0.306 | 102 |
| YesCollegeDegree | -0.18 | 0.13 | -0.10 | 0.07 | -0.44 – 0.08 | -1.34 | 0.183 | 102 |
| time [1] × GroupID [0.5] | -0.09 | 0.10 | -0.12 | 0.13 | -0.28 – 0.10 | -0.95 | 0.343 | 201 |
| time [2] × GroupID [0.5] | -0.03 | 0.08 | -0.04 | 0.10 | -0.18 – 0.12 | -0.39 | 0.695 | 201 |

| Random Effects | |
| --- | --- |
| σ^2^ | 0.06 |
| τ_00_ _SubID_ | 0.28 |
| τ_11_ _SubID.time1_ | 0.21 |
| τ_11_ _SubID.time2_ | 0.10 |
| ρ_01_ | 0.07 |
|  | -0.07 |
| ICC | 0.88 |
| N _SubID_ | 108 |
| Observations | 313 |
| Marginal R^2^ / Conditional R^2^ | 0.087 / 0.894 |

*Note*. This random intercept model shows the change in inclusion of other in the self for a stranger (IOS; Aron et al., 1992) from T1 to T2 and T2 to T3. Time is a factor with three levels (T1, T2, T3). GroupID is centered (LKM = 0.5, PMR = -0.5). Demographics are dummy-coded (gender reference group female, education reference group no college degree). We report standardized betas for all regressions.

**Changes in Inclusion of Other in the Self**

We observed an increase in average IOS (std. β = 0.15, SE = 0.07, t(201) = 2.21, 95% CI [0.02, 0.29], p = .028) and IOS for a stranger from T1 to T2 (std. β =0.16, SE = 0.07, t(201) = 2.29, 95% CI [0.03, 0.40], p = .023) across groups (Table 5 & 6; Figure 2).

**Figure 2**

*Significant Changes in Inclusion of Other in the Self (IOS) Over Time for Both Groups (N = 108)*


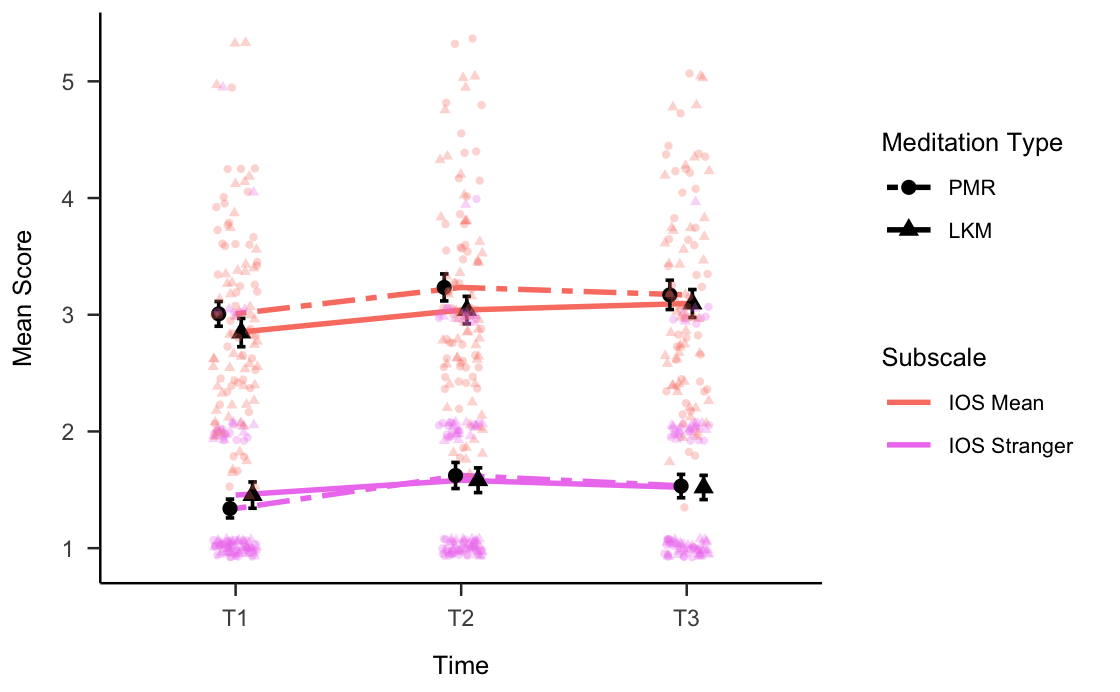


*Note.* IOS Mean and Stranger increased after the intervention across meditation groups. We ran separate multilevel models (one per subscale in addition to average IOS) with group, time, and IOS mean and stranger as predictors and there was an increase in mean IOS (IOS; Aron et al., 1992) from T1 to T2 (β = 0.15, SE = 0.07, 201) = 2.21, 95% CI [0.02, 0.29], p = .028). No further change was observed from T2 to T3 (β = 0.04, SE = 0.07, 201) = 0.66, 95% *CI* [-0.08, 0.17], p = .509). No main effect of the intervention on IOS was found (β = -0.10, SE = 0.16, 102) = -0.59, 95% *CI* [-0.41, 0.22], p = .558), nor were there any statistically significant interactions between time and intervention. IOS with strangers increased from T1 to T2 (β = 0.21, SE = 0.09, 201) = 2.29, 95% *CI* [0.03, 0.40], p = .023), indicating that participants in both groups felt more connected to strangers after the intervention.

### ***Bayes Factors for Null Findings: Mixed Models Group Differences Over Time***

To formally evaluate evidence for the absence of null effects we interpret, we computed Bayes factors (BFs) for predictors using their corresponding *t-statistic*s. This was done using the ttest.tstat() function from the BayesFactor R package (Morey & Rouder, 2011), which implements the method proposed by Rouder et al. (2009). This approach applies the Jeffreys–Zellner–Siow (JZS) prior, a default Cauchy distribution centered at zero with a scale parameter of r = 0.707. We report BF01​, the ratio of evidence in favor of the null hypothesis over the alternative. Bayes factor interpretation followed common conventions: BF01 between 1–3 indicates anecdotal evidence for H₀, 3–10 moderate evidence, 10–30 strong evidence, 30–100 very strong evidence, and >100 extreme evidence for H_01_ (Jeffreys, 1961; Schmalz et al., 2023).

| **Table 7**  *Loneliness: Conversion of t-statistics into Bayes Factors* |
| --- |

| *Null Effect of Interest* | *t* | *df* | *BF10* | *BF01* | *Interpretation* |
| --- | --- | --- | --- | --- | --- |
| time[2] | -1.01 | 201 | 0.130 | 7.701 | Moderate evidence for H0 |
| GroupID [0.5] | 1.86 | 102 | 0.573 | 1.747 | Anecdotal evidence for H0 |
| time [1] × GroupID [0.5] | 1.16 | 201 | 0.152 | 6.565 | Moderate evidence for H0 |
| time [2] × GroupID [0.5] | 0.62 | 201 | 0.095 | 10.524 | Strong evidence for H0 |

*Note*. Bayes Factors provide moderate to strong evidence for the null hypothesis to be true for the change in loneliness from T2-T3 and for the interaction effects suggesting that the two intervention groups did not differ in loneliness over time. Moreover, we found anecdotal evidence for the null regarding the main effect of group on loneliness, making this evidence inconclusive.

| **Table 8**  *Trait Empathic Concern (EC): Conversion of t-statistics into Bayes Factors* |
| --- |

| *Null Effect of Interest* | *t* | *df* | *BF_10_* | *BF_01_* | *Interpretation* |
| --- | --- | --- | --- | --- | --- |
| time[1] | -0.12 | 201 | 0.079 | 12.63 | Strong evidence for H0 |
| time[2] | -1.00 | 201 | 0.129 | 7.777 | Moderate evidence for H0 |
| GroupID [0.5] | -0.29 | 102 | 0.114 | 8.795 | Moderate evidence for H0 |
| time [1] × GroupID [0.5] | 1.10 | 201 | 0.143 | 7.016 | Moderate evidence for H0 |
| time [2] × GroupID [0.5] | 1.18 | 201 | 0.156 | 6.417 | Moderate evidence for H0 |

*Note*. Bayes Factors provide moderate to strong evidence for the null hypothesis to be true for the change in trait empathic concern from T2-T3 and for the interaction effects suggesting that the two intervention groups did not differ in trait empathic concern over time. Moreover, we found moderate evidence for a null effect for group as the main effect, indicating that the groups were overall similar in trait empathic concern.

| **Table 9**  *Trait Perspective Taking (PT): Conversion of t-statistics into Bayes Factors* |
| --- |

| *Null Effect of Interest* | *t* | *df* | *BF_10_* | *BF_01_* | *Interpretation* |
| --- | --- | --- | --- | --- | --- |
| time[1] | 1.04 | 201 | 0.134 | 7.472 | Moderate evidence for H0 |
| time[2] | 0.13 | 201 | 0.079 | 12.612 | Strong evidence for H0 |
| GroupID [0.5] | -0.02 | 102 | 0.109 | 9.161 | Moderate evidence for H0 |
| time [1] × GroupID [0.5] | 0.64 | 201 | 0.096 | 10.395 | Strong evidence for H0 |
| time [2] × GroupID [0.5] | 0.68 | 201 | 0.099 | 10.128 | Strong evidence for H0 |

*Note*. Bayes Factors provide moderate to strong evidence for the null hypothesis to be true for the change in trait empathic concern from T2-T3 and for the interaction effects suggesting that the two intervention groups did not differ in trait empathic concern over time. Moreover, we found moderate evidence for a null effect for group as the main effect, indicating that the groups were overall similar in trait empathic concern.

### ***Post-hoc Sensitivity Analysis: Mixed Models Group Differences Over Time***

To define the smallest effect size of interest (SESOI; Lakens et al., 2018), we relied on prior empirical work. A meta-analysis (Teoh et al., 2021) synthesizing results from eight randomized controlled trials (*N* = 815) found that mindfulness-based interventions yielded a standardized mean difference (SMD) of −0.36 [−0.70, −0.03] in reducing loneliness. Based on this and conventional benchmarks in psychological science (Cohen, 1988), we defined a SESOI of *d* = 0.30—representing a small but practically meaningful improvement in loneliness. Because we also examined trait empathy as a secondary outcome, we selected the same SESOI of *d* = 0.30, consistent with a conservative estimate drawn from the Kreplin et al. (2018) meta-analysis of meditation’s effects on empathy (*r* = 0.44, *95% CI* [0.03, 0.84]). A post-hoc power analysis using G*Power (Faul et al., 2007) indicated that, in our two-sided design (2 groups, 3 repeated measures, correlation among measures = 0.5, *ε* = 1), a total sample size of only 20 participants would be sufficient to detect such an effect with 80% power at *α* = .05; our sample of 108 therefore provided ample power to detect this effect.

To complement this, we conducted a post hoc sensitivity analysis to determine the smallest effect our actual sample size could reliably detect. Using G*Power (Faul et al., 2007), we found that our behavioral sample of 108 participants provided 80% power to detect an effect size of *f* = 0.122, equivalent to Cohen’s *d* ≈ 0.24 (a small-to-moderate effect), in a repeated-measures ANOVA with a within-between interaction. A post hoc power analysis further confirmed that, given our design and sample size, we had >99% power to detect effects of *d* = 0.30. This is a conservative estimate, as linear mixed-effects models—used in our primary analyses—are generally more efficient and powerful than standard ANOVA approaches (Judd et al., 2001; Westfall et al., 2014). Given this, we suggest that the nonsignificant results observed (particularly for the time x group interactions) are unlikely to be due to limited power. Together with Bayes factors showing moderate-to-strong evidence for the null for most findings, our results support the interpretation that these effects are likely absent rather than undetected.

## **Supplemental Information C: State Empathy for Fear/Unpleasantness but Not Pain Predicts Loneliness**

### ***Differences in Subjective Ratings***

**Table 10**

Group Differences in Subjective Ratings of Pain and Fear

| *Measure* | *LKM (M±SD)* | *PMR (M±SD)* | *t* | *p* | *d* | *95% CI* |
| --- | --- | --- | --- | --- | --- | --- |
| Pounds per Square Inch (PSI) | 32.07 ± 13.33 | 34.60 ± 15.41 | 0.647 | .520 | 0.177 | [-5.31, 10.38] |
| Pain Rating (Self) | 4.17 ± 1.38 | 4.18 ± 0.95 | 0.023 | .982 | 0.006 | [-0.65, 0.67] |
| Fear of Pain (Self) | 3.17 ± 1.39 | 3.40 ± 1.38 | 0.601 | .551 | 0.164 | [-0.53, 0.99] |
| Pain Unpleasantness (Self) | 3.45 ± 1.18 | 3.96 ± 1.57 | 1.365 | .178 | 0.373 | [-0.24, 1.26] |
| Pain Rating (Other) | 4.07 ± 1.22 | 4.04 ± 1.27 | -0.085 | .932 | 0.023 | [-0.71, 0.65] |
| Fear of Pain (Other) | 2.93 ± 1.39 | 3.32 ± 1.52 | 0.983 | .330 | 0.268 | [-0.41, 1.18] |
| Pain Unpleasantness (Other) | 3.45 ± 1.45 | 3.64 ± 1.38 | 0.495 | .623 | 0.135 | [-0.59, 0.97] |

*Note*. *M* = mean, *SD* = standard deviation, *t* = t-statistic, *p* = p-value, *d* = Cohen’s *d*, CI = 95% Confidence Interval. Pounds per square inch (PSI) indicates the level of pressure participants perceived to be a 13.5 (“slightly intense” on the 21-point Gracely Box Scale; Gracely et al., 1992). In the scanner, participants indicated how painful and outside of the scanner they indicated how unpleasant the pressure felt to themselves and their study partner respectively, as well as how fearful/anxious they felt during anticipation of the painful stimulus. These ratings were done on a 7-point scale (1: No pain/unpleasantness/fear to 7: Extreme pain/unpleasantness/fear).

### ***Study Partner Perceptions***

**Table 11**

***Study Partner Perception Descriptives and Group Differences***

| *Variable* | *M LKM* | *M PMR* | *SD LKM* | *SD PMR* | *t* | *df* | *p* | *95%CI_lower* | *95%CI_upper* | *d* |
| --- | --- | --- | --- | --- | --- | --- | --- | --- | --- | --- |
| IOS Mean | 3.04 | 3.29 | 0.93 | 0.85 | 1.01 | 52 | 0.32 | -0.24 | 0.74 | 0.28 |
| IOS Stranger | 1.72 | 1.64 | 0.88 | 0.76 | -0.37 | 52 | 0.71 | -0.54 | 0.37 | 0.10 |
| IOS Study Partner | 2.38 | 1.84 | 1.12 | 1.31 | -1.63 | 52 | 0.11 | -1.20 | 0.12 | 0.45 |
| Liking Study Partner | 5.00 | 4.96 | 1.07 | 1.27 | -0.13 | 52 | 0.90 | -0.68 | 0.60 | 0.03 |
| Real Study Partner | 5.72 | 5.4 | 1.85 | 1.55 | -0.69 | 52 | 0.49 | -1.27 | 0.62 | 0.19 |

*Note*. *M* = mean, *SD* = standard deviation, *t* = t-statistic, *p* = p-value, *d* = Cohen’s *d*, CI = 95% Confidence Interval. IOS = Inclusion of Other in the Self (Aron et al., 1992) for a stranger to compare with the study partner, who also was a stranger to the participants. Participants were also asked how much they liked the study partner and whether they thought the study partner was real.

## **Supplemental Information D: State Empathy for Fear/Unpleasantness but Not Pain Predicts Loneliness**

**Figure 2**

*Regressions Loneliness and State Empathy (N = 54)*


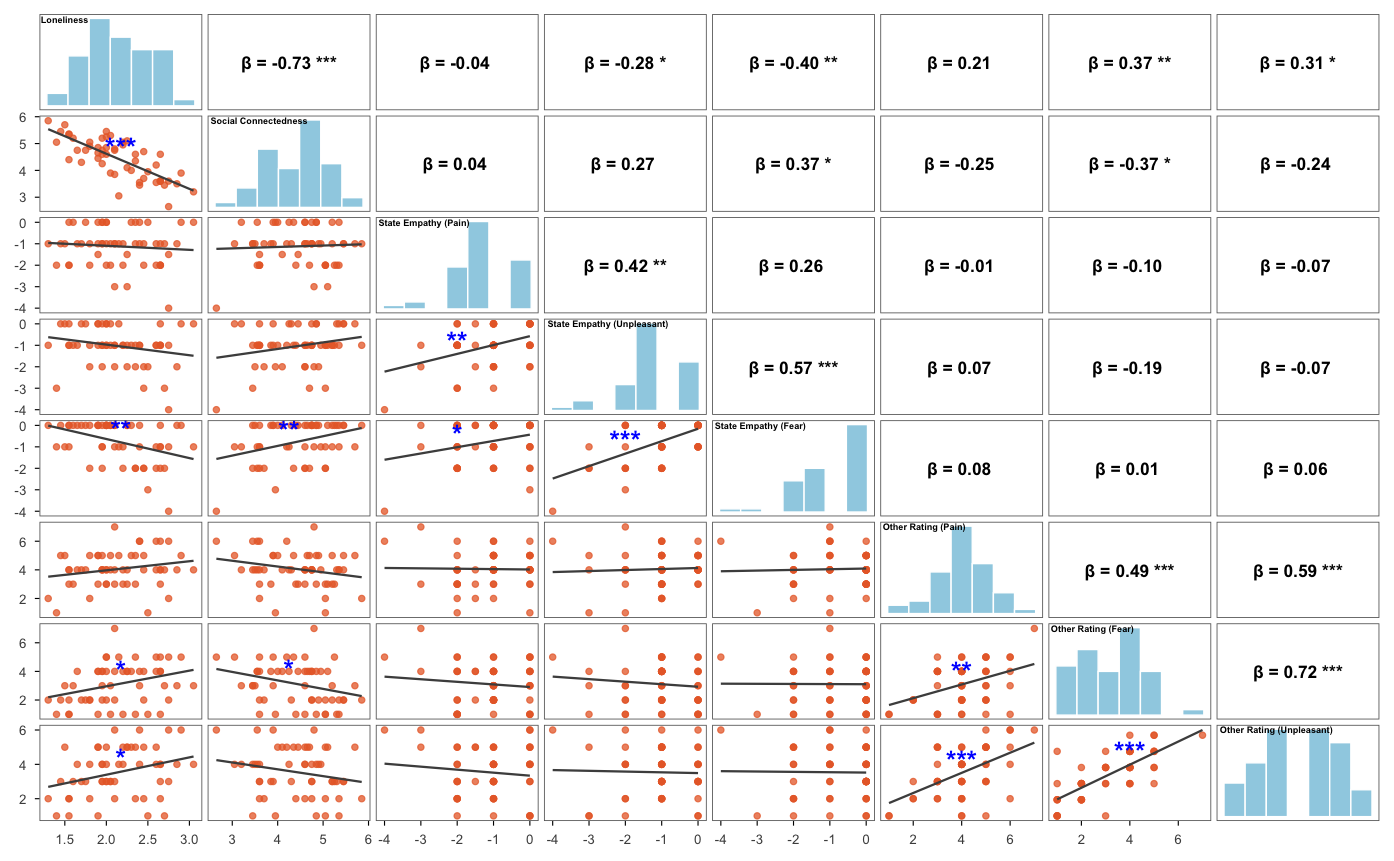


*Note*. Beta from regressions of state empathy, self, and other ratings with loneliness and social connectedness after controlling for covariates age, gender, education, and group.

***Frequentist Linear Regressions State Empathy (Fear)***

**Table 12**

Loneliness ~ stateEmp_fear + GroupID + age + Male + Other + YesCollegeDegree

| *Predictors* | *b* | | *SE* | | *std. B* | | *std. SE* | | *95% CI* | | | *t* | | *p* | | *df* | |
| --- | --- | --- | --- | --- | --- | --- | --- | --- | --- | --- | --- | --- | --- | --- | --- | --- | --- |
| (Intercept) | 2.12 | 0.17 | | -0.25 | | 0.18 | | 1.78 – 2.46 | | | 12.42 | | **<0.001** | | 47.00 | | |
| age | -0.00 | 0.00 | | -0.11 | | 0.14 | | -0.01 – 0.01 | | | -0.83 | | 0.410 | | 47.00 | | |
| Male | -0.05 | 0.11 | | -0.05 | | 0.13 | | -0.27 – 0.18 | | | -0.40 | | 0.690 | | 47.00 | | |
| Other | 0.47 | 0.29 | | 0.21 | | 0.13 | | -0.11 – 1.04 | | | 1.63 | | 0.110 | | 47.00 | | |
| YesCollegeDegree | -0.11 | 0.14 | | -0.11 | | 0.14 | | -0.41 – 0.18 | | | -0.79 | | 0.434 | | 47.00 | | |
| GroupID [LKM] | 0.20 | 0.11 | | 0.47 | | 0.25 | | -0.02 – 0.42 | | | 1.85 | | 0.071 | | 47.00 | | |
| stateEmp fear | -0.18 | 0.06 | | -0.40 | | 0.13 | | -0.29 – -0.06 | | | -3.10 | | **0.003** | | 47.00 | | |
| Observations | 54 | | | | | | | | | | | | | | | |  |
| R^2^ / R^2^ adjusted | 0.292 / 0.202 | | | | | | | | |  | | | | | | | |
| AIC | 58.093 | | | | | | | | | | | | | | | |  |

*Note*. Linear regression showing the effect of state empathy (fear) on trait loneliness (UCLA; Russell, 1996).

**Table 13**

Social Connectedness ~ stateEmp_fear + GroupID + age + Male + Other + YesCollegeDegree

| *Predictors* | *b* | | *SE* | | *std. B* | | *std. SE* | | *95% CI* | | *t* | *p* | | *df* | | |
| --- | --- | --- | --- | --- | --- | --- | --- | --- | --- | --- | --- | --- | --- | --- | --- | --- |
| (Intercept) | 4.75 | 0.32 | | 0.11 | | 0.20 | | 4.10 – 5.39 | | | 14.81 | **<0.001** | 47.00 | | |  |
| age | 0.00 | 0.01 | | 0.06 | | 0.15 | | -0.01 – 0.02 | | | 0.40 | 0.694 | 47.00 | | |  |
| Male | -0.08 | 0.21 | | -0.06 | | 0.14 | | -0.51 – 0.34 | | | -0.39 | 0.695 | 47.00 | | |  |
| Other | -0.64 | 0.54 | | -0.17 | | 0.14 | | -1.72 – 0.44 | | | -1.19 | 0.240 | 47.00 | | |  |
| YesCollegeDegree | -0.09 | 0.27 | | -0.05 | | 0.15 | | -0.63 – 0.46 | | | -0.32 | 0.747 | 47.00 | | |  |
| GroupID [LKM] | -0.15 | 0.20 | | -0.20 | | 0.27 | | -0.56 – 0.26 | | | -0.72 | 0.473 | 47.00 | | |  |
| stateEmp fear | 0.28 | 0.11 | | 0.37 | | 0.14 | | 0.07 – 0.50 | | | 2.64 | **0.011** | 47.00 | | |  |
| Observations | 54 | | | | | | | | | | | | | |  | |
| R^2^ / R^2^ adjusted | 0.163 / 0.056 | | | | | | | | |  | | | | | | |
| AIC | 126.055 | | | | | | | | | | | | | |  | |

*Note*. Linear regression showing the effect of state empathy (fear) on trait social connectedness (SCS-R; Lee et al., 2001).

**Table 14**

Trait Empathic Concern (IRI) ~ stateEmp_fear + GroupID + age + Male + Other + YesCollegeDegree

| *Predictors* | *b* | *SE* | *std. B* | *std. SE* | *95% CI* | | *t* | *p* | *df* | |
| --- | --- | --- | --- | --- | --- | --- | --- | --- | --- | --- |
| (Intercept) | 4.28 | 0.27 | 0.15 | 0.21 | 3.75 – 4.82 | | 16.14 | **<0.001** | 47.00 | |
| age | 0.00 | 0.01 | 0.01 | 0.16 | -0.01 – 0.01 | | 0.03 | 0.974 | 47.00 | |
| Male | -0.28 | 0.17 | -0.24 | 0.15 | -0.63 – 0.07 | | -1.63 | 0.110 | 47.00 | |
| Other | -0.45 | 0.45 | -0.14 | 0.14 | 3.75 – 4.82 | | -1.00 | 0.322 | 47.00 | |
| YesCollegeDegree | 0.02 | 0.23 | 0.01 | 0.16 | -0.01 – 0.01 | | 0.07 | 0.941 | 47.00 | |
| GroupID [LKM] | -0.16 | 0.17 | -0.28 | 0.29 | -0.63 – 0.07 | | -0.97 | 0.339 | 47.00 | |
| stateEmp fear | -0.02 | 0.09 | -0.04 | 0.14 | 3.75 – 4.82 | | -0.25 | 0.805 | 47.00 | |
| Observations | 54 | | | | | | | | |  |
| R^2^ / R^2^ adjusted | 0.098 / -0.018 | | | | |  | | | | |
| AIC | 105.759 | | | | | | | | |  |

*Note*. Linear regression showing the effect of state empathy (fear) on trait empathic concern (IRI; Davis, 1983).

**Table 15**

Trait Perspective Taking (IRI) ~ stateEmp_fear + GroupID + age + Male + Other + YesCollegeDegree

| *Predictors* | *b* | *SE* | *std. B* | *std. SE* | *95% CI* | | *t* | *p* | *df* | |
| --- | --- | --- | --- | --- | --- | --- | --- | --- | --- | --- |
| (Intercept) | 4.15 | 0.30 | 0.12 | 0.21 | 3.53 – 4.76 | | 13.62 | **<0.001** | 47.00 | |
| age | -0.01 | 0.01 | -0.27 | 0.16 | -0.03 – 0.00 | | -1.71 | 0.094 | 47.00 | |
| Male | 0.06 | 0.20 | 0.05 | 0.15 | -0.34 – 0.46 | | 0.31 | 0.755 | 47.00 | |
| Other | 0.01 | 0.51 | 0.00 | 0.15 | -1.02 – 1.04 | | 0.02 | 0.984 | 47.00 | |
| YesCollegeDegree | 0.16 | 0.26 | 0.10 | 0.16 | -0.36 – 0.68 | | 0.61 | 0.543 | 47.00 | |
| GroupID [LKM] | -0.15 | 0.19 | -0.22 | 0.29 | -0.54 – 0.24 | | -0.77 | 0.444 | 47.00 | |
| stateEmp fear | -0.03 | 0.10 | -0.04 | 0.15 | -0.24 – 0.18 | | -0.29 | 0.776 | 47.00 | |
| Observations | 54 | | | | | | | | |  |
| R^2^ / R^2^ adjusted | 0.069 / -0.050 | | | | |  | | | | |
| AIC | 120.558 | | | | | | | | |  |

*Note*. Linear regression showing the effect of state empathy (fear) on trait perspective taking (IRI; Davis, 1983).

**Table 16**

Mean IOS ~ stateEmp_fear + GroupID + age + Male + Other + YesCollegeDegree

| *Predictors* | *b* | *SE* | *std. B* | *std. SE* | *95% CI* | | *t* | *p* | *df* | |
| --- | --- | --- | --- | --- | --- | --- | --- | --- | --- | --- |
| (Intercept) | 3.89 | 0.37 | 0.17 | 0.19 | 3.14 – 4.64 | | 10.46 | **<0.001** | 47.00 | |
| age | -0.02 | 0.01 | -0.29 | 0.14 | -0.04 – -0.00 | | -2.03 | **0.048** | 47.00 | |
| Male | 0.41 | 0.24 | 0.23 | 0.14 | -0.08 – 0.90 | | 1.67 | 0.102 | 47.00 | |
| Other | 0.89 | 0.62 | 0.19 | 0.13 | -0.36 – 2.15 | | 1.43 | 0.159 | 47.00 | |
| YesCollegeDegree | 0.08 | 0.32 | 0.04 | 0.15 | -0.56 – 0.71 | | 0.24 | 0.810 | 47.00 | |
| GroupID [LKM] | -0.28 | 0.24 | -0.31 | 0.26 | -0.75 – 0.20 | | -1.17 | 0.247 | 47.00 | |
| stateEmp fear | 0.22 | 0.13 | 0.24 | 0.13 | -0.03 – 0.47 | | 1.78 | 0.082 | 47.00 | |
| Observations | 54 | | | | | | | | |  |
| R^2^ / R^2^ adjusted | 0.229 / 0.131 | | | | |  | | | | |
| AIC | 142.076 | | | | | | | | |  |

*Note*. Linear regression showing the effect of state empathy (fear) on mean inclusion of other in the self (IOS; Aron et al., 1992).

**Table 17**

IOS Stranger ~ stateEmp_fear + GroupID + age + Male + Other + YesCollegeDegree

| *Predictors* | *b* | *SE* | *std. B* | *std. SE* | *95% CI* | | *t* | *p* | *df* | |
| --- | --- | --- | --- | --- | --- | --- | --- | --- | --- | --- |
| (Intercept) | 1.91 | 0.33 | 0.04 | 0.18 | 1.25 – 2.56 | | 5.83 | **<0.001** | 47.00 | |
| age | -0.02 | 0.01 | -0.35 | 0.14 | -0.04 – -0.00 | | -2.51 | **0.016** | 47.00 | |
| Male | 0.53 | 0.21 | 0.32 | 0.13 | 0.10 – 0.96 | | 2.47 | **0.017** | 47.00 | |
| Other | 0.26 | 0.55 | 0.06 | 0.13 | -0.84 – 1.37 | | 0.48 | 0.635 | 47.00 | |
| YesCollegeDegree | 0.60 | 0.28 | 0.31 | 0.14 | 0.04 – 1.16 | | 2.16 | **0.036** | 47.00 | |
| GroupID [LKM] | -0.06 | 0.21 | -0.07 | 0.25 | -0.48 – 0.36 | | -0.28 | 0.778 | 47.00 | |
| stateEmp fear | 0.20 | 0.11 | 0.23 | 0.13 | -0.02 – 0.42 | | 1.81 | 0.076 | 47.00 | |
| Observations | 54 | | | | | | | | |  |
| R^2^ / R^2^ adjusted | 0.292 / 0.201 | | | | |  | | | | |
| AIC | 128.185 | | | | | | | | |  |

*Note*. Linear regression showing the effect of state empathy (fear) on inclusion of other in the self for a stranger (IOS; Aron et al., 1992).

**Table 18**

IOS Study Partner ~ stateEmp_fear + GroupID + age + Male + Other + YesCollegeDegree

| *Predictors* | *b* | *SE* | *std. B* | *std. SE* | *95% CI* | | *t* | *p* | *df* | |
| --- | --- | --- | --- | --- | --- | --- | --- | --- | --- | --- |
| (Intercept) | 2.25 | 0.55 | -0.21 | 0.20 | 1.14 – 3.35 | | 4.10 | **<0.001** | 47.00 | |
| age | -0.01 | 0.01 | -0.15 | 0.15 | -0.04 – 0.01 | | -0.99 | 0.329 | 47.00 | |
| Male | 0.29 | 0.36 | 0.12 | 0.15 | -0.43 – 1.02 | | 0.81 | 0.422 | 47.00 | |
| Other | -0.44 | 0.92 | -0.07 | 0.14 | -2.29 – 1.41 | | -0.48 | 0.636 | 47.00 | |
| YesCollegeDegree | 0.17 | 0.46 | 0.06 | 0.16 | -0.76 – 1.11 | | 0.37 | 0.711 | 47.00 | |
| GroupID [LKM] | 0.49 | 0.35 | 0.40 | 0.28 | -0.21 – 1.19 | | 1.42 | 0.163 | 47.00 | |
| stateEmp fear | 0.18 | 0.18 | 0.14 | 0.14 | -0.19 – 0.55 | | 0.97 | 0.336 | 47.00 | |
| Observations | 54 | | | | | | | | |  |
| R^2^ / R^2^ adjusted | 0.113 / -0.000 | | | | |  | | | | |
| AIC | 184.049 | | | | | | | | |  |

*Note*. Linear regression showing the effect of state empathy (fear) on inclusion of other in the self for the study partner (IOS; Aron et al., 1992).

### ***Frequentist Linear Regressions State Empathy (Unpleasantness)***

**Table 19**

Loneliness ~ stateEmp_unpleasant + GroupID + age + Male + Other + YesCollegeDegree

| *Predictors* | *b* | | *SE* | | *std. B* | | *std. SE* | | *95% CI* | | | *t* | | *p* | | *df* | |
| --- | --- | --- | --- | --- | --- | --- | --- | --- | --- | --- | --- | --- | --- | --- | --- | --- | --- |
| (Intercept) | 2.20 | 0.18 | | -0.25 | | 0.19 | | 1.84 – 2.55 | | | 12.53 | | **<0.001** | | 47.00 | | |
| age | -0.00 | 0.00 | | -0.15 | | 0.15 | | -0.01 – 0.00 | | | -1.05 | | 0.301 | | 47.00 | | |
| Male | -0.12 | 0.11 | | -0.14 | | 0.13 | | -0.35 – 0.11 | | | -1.07 | | 0.290 | | 47.00 | | |
| Other | 0.46 | 0.30 | | 0.21 | | 0.13 | | -0.14 – 1.07 | | | 1.53 | | 0.132 | | 47.00 | | |
| YesCollegeDegree | -0.11 | 0.15 | | -0.11 | | 0.15 | | -0.42 – 0.19 | | | -0.75 | | 0.456 | | 47.00 | | |
| GroupID [LKM] | 0.20 | 0.11 | | 0.47 | | 0.26 | | -0.02 – 0.43 | | | 1.79 | | 0.079 | | 47.00 | | |
| stateEmp painUnpleasant | -0.13 | 0.06 | | -0.28 | | 0.13 | | -0.26 – -0.01 | | | -2.13 | | **0.038** | | 47.00 | | |
| Observations | 54 | | | | | | | | | | | | | | | |  |
| R^2^ / R^2^ adjusted | 0.222 / 0.123 | | | | | | | | |  | | | | | | | |
| AIC | 63.178 | | | | | | | | | | | | | | | |  |

*Note*. Linear regression showing the effect of state empathy (unpleasant) on trait loneliness (UCLA; Russell, 1996).

**Table 20**

*S*ocial Connectedness ~ stateEmp_unpleasant + GroupID + age + Male + Other + YesCollegeDegree

| *Predictors* | *b* | | *SE* | | *std. B* | | *std. SE* | | *95% CI* | | *t* | *p* | | *df* | | |
| --- | --- | --- | --- | --- | --- | --- | --- | --- | --- | --- | --- | --- | --- | --- | --- | --- |
| (Intercept) | 4.63 | 0.32 | | 0.11 | | 0.20 | | 3.98 – 5.28 | | | 14.27 | **<0.001** | 47.00 | | |  |
| age | 0.01 | 0.01 | | 0.09 | | 0.16 | | -0.01 – 0.02 | | | 0.61 | 0.547 | 47.00 | | |  |
| Male | 0.04 | 0.21 | | 0.03 | | 0.14 | | -0.38 – 0.46 | | | 0.19 | 0.853 | 47.00 | | |  |
| Other | -0.63 | 0.56 | | -0.16 | | 0.14 | | -1.75 – 0.49 | | | -1.13 | 0.262 | 47.00 | | |  |
| YesCollegeDegree | -0.09 | 0.28 | | -0.05 | | 0.16 | | -0.65 – 0.48 | | | -0.31 | 0.757 | 47.00 | | |  |
| GroupID [LKM] | -0.15 | 0.21 | | -0.21 | | 0.28 | | -0.57 – 0.27 | | | -0.72 | 0.472 | 47.00 | | |  |
| stateEmp painUnpleasant | 0.22 | 0.12 | | 0.27 | | 0.14 | | -0.02 – 0.45 | | | 1.87 | 0.068 | 47.00 | | |  |
| Observations | 54 | | | | | | | | | | | | | |  | |
| R^2^ / R^2^ adjusted | 0.105 / -0.009 | | | | | | | | |  | | | | | | |
| AIC | 129.651 | | | | | | | | | | | | | |  | |

*Note*. Linear regression showing the effect of state empathy (unpleasant) on trait social connectedness (SCS-R; Lee et al., 2001).

**Table 21**

Trait Empathic Concern (IRI) ~ stateEmp_unpleasant + GroupID + age + Male + Other + YesCollegeDegree

| *Predictors* | *b* | *SE* | *std. B* | *std. SE* | *95% CI* | | *t* | *p* | *df* | |
| --- | --- | --- | --- | --- | --- | --- | --- | --- | --- | --- |
| (Intercept) | 4.35 | 0.26 | 0.14 | 0.20 | 3.83 – 4.87 | | 16.88 | **<0.001** | 47.00 | |
| age | 0.00 | 0.01 | 0.02 | 0.16 | -0.01 – 0.01 | | 0.15 | 0.882 | 47.00 | |
| Male | -0.30 | 0.17 | -0.26 | 0.14 | -0.64 – 0.03 | | -1.82 | 0.075 | 47.00 | |
| Other | -0.51 | 0.44 | -0.16 | 0.14 | -1.40 – 0.38 | | -1.14 | 0.259 | 47.00 | |
| YesCollegeDegree | 0.04 | 0.22 | 0.03 | 0.16 | -0.41 – 0.49 | | 0.17 | 0.869 | 47.00 | |
| GroupID [LKM] | -0.15 | 0.17 | -0.26 | 0.28 | -0.49 – 0.19 | | -0.90 | 0.371 | 47.00 | |
| stateEmp painUnpleasant | 0.09 | 0.09 | 0.13 | 0.14 | -0.10 – 0.27 | | 0.94 | 0.352 | 47.00 | |
| Observations | 54 | | | | | | | | |  |
| R^2^ / R^2^ adjusted | 0.113 / -0.000 | | | | |  | | | | |
| AIC | 104.825 | | | | | | | | |  |

*Note*. Linear regression showing the effect of state empathy (unpleasant) on trait empathic concern (IRI; Davis, 1983).

**Table 22**

Trait Perspective Taking (IRI) ~ stateEmp_unpleasant + GroupID + age + Male + Other + YesCollegeDegree

| *Predictors* | *b* | *SE* | *std. B* | *std. SE* | *95% CI* | | *t* | *p* | *df* | |
| --- | --- | --- | --- | --- | --- | --- | --- | --- | --- | --- |
| (Intercept) | 4.19 | 0.30 | 0.11 | 0.21 | 3.59 – 4.79 | | 14.07 | **<0.001** | 47.00 | |
| age | -0.01 | 0.01 | -0.26 | 0.16 | -0.03 – 0.00 | | -1.65 | 0.106 | 47.00 | |
| Male | 0.04 | 0.19 | 0.03 | 0.15 | -0.35 – 0.43 | | 0.23 | 0.822 | 47.00 | |
| Other | -0.03 | 0.51 | -0.01 | 0.15 | -1.06 – 1.00 | | -0.05 | 0.958 | 47.00 | |
| YesCollegeDegree | 0.17 | 0.26 | 0.11 | 0.16 | -0.35 – 0.69 | | 0.66 | 0.512 | 47.00 | |
| GroupID [LKM] | -0.14 | 0.19 | -0.21 | 0.29 | -0.53 – 0.25 | | -0.73 | 0.467 | 47.00 | |
| stateEmp painUnpleasant | 0.04 | 0.11 | 0.06 | 0.14 | -0.17 – 0.25 | | 0.38 | 0.702 | 47.00 | |
| Observations | 54 | | | | | | | | |  |
| R^2^ / R^2^ adjusted | 0.070 / -0.048 | | | | |  | | | | |
| AIC | 120.481 | | | | | | | | |  |

*Note*. Linear regression showing the effect of state empathy (unpleasant) on trait perspective taking (IRI; Davis, 1983).

**Table 23**

Mean IOS ~ stateEmp_unpleasant + GroupID + age + Male + Other + YesCollegeDegree

| *Predictors* | *b* | *SE* | *std. B* | *std. SE* | *95% CI* | | *t* | *p* | *df* | |
| --- | --- | --- | --- | --- | --- | --- | --- | --- | --- | --- |
| (Intercept) | 3.70 | 0.38 | 0.18 | 0.20 | 2.94 – 4.45 | | 9.84 | **<0.001** | 47.00 | |
| age | -0.02 | 0.01 | -0.29 | 0.15 | -0.04 – 0.00 | | -1.96 | 0.056 | 47.00 | |
| Male | 0.52 | 0.24 | 0.29 | 0.14 | 0.03 – 1.01 | | 2.14 | **0.038** | 47.00 | |
| Other | 1.00 | 0.65 | 0.21 | 0.14 | -0.30 – 2.30 | | 1.55 | 0.128 | 47.00 | |
| YesCollegeDegree | 0.04 | 0.33 | 0.02 | 0.15 | -0.61 – 0.70 | | 0.13 | 0.899 | 47.00 | |
| GroupID [LKM] | -0.30 | 0.24 | -0.34 | 0.27 | -0.79 – 0.19 | | -1.23 | 0.224 | 47.00 | |
| stateEmp painUnpleasant | -0.01 | 0.13 | -0.01 | 0.14 | -0.28 – 0.26 | | -0.07 | 0.946 | 47.00 | |
| Observations | 54 | | | | | | | | |  |
| R^2^ / R^2^ adjusted | 0.178 / 0.073 | | | | |  | | | | |
| AIC | 145.579 | | | | | | | | |  |

*Note*. Linear regression showing the effect of state empathy (unpleasant) on mean inclusion of other in the self (IOS; Aron et al., 1992).

**Table 24**

IOS Stranger ~ stateEmp_unpleasant + GroupID + age + Male + Other + YesCollegeDegree

| *Predictors* | *b* | *SE* | *std. B* | *std. SE* | *95% CI* | | *t* | *p* | *df* | |
| --- | --- | --- | --- | --- | --- | --- | --- | --- | --- | --- |
| (Intercept) | 1.79 | 0.33 | 0.05 | 0.19 | 1.13 – 2.45 | | 5.43 | **<0.001** | 47.00 | |
| age | -0.02 | 0.01 | -0.33 | 0.14 | -0.04 – -0.00 | | -2.33 | **0.024** | 47.00 | |
| Male | 0.62 | 0.21 | 0.38 | 0.13 | 0.19 – 1.05 | | 2.91 | **0.005** | 47.00 | |
| Other | 0.30 | 0.57 | 0.07 | 0.13 | -0.83 – 1.44 | | 0.54 | 0.593 | 47.00 | |
| YesCollegeDegree | 0.59 | 0.29 | 0.30 | 0.15 | 0.01 – 1.16 | | 2.06 | **0.045** | 47.00 | |
| GroupID [LKM] | -0.07 | 0.21 | -0.08 | 0.26 | -0.50 – 0.36 | | -0.33 | 0.745 | 47.00 | |
| stateEmp painUnpleasant | 0.09 | 0.12 | 0.10 | 0.13 | -0.15 – 0.32 | | 0.75 | 0.457 | 47.00 | |
| Observations | 54 | | | | | | | | |  |
| R^2^ / R^2^ adjusted | 0.251 / 0.156 | | | | |  | | | | |
| AIC | 131.201 | | | | | | | | |  |

*Note*. Linear regression showing the effect of state empathy (unpleasant) on inclusion of other in the self for a stranger (IOS; Aron et al., 1992).

**Table 25**

*IOS Study Partner ~ stateEmp_unpleasant + GroupID + age + Male +* Other + YesCollegeDegree

| *Predictors* | *b* | *SE* | *std. B* | *std. SE* | *95% CI* | | *t* | *p* | *df* | |
| --- | --- | --- | --- | --- | --- | --- | --- | --- | --- | --- |
| (Intercept) | 2.07 | 0.54 | -0.20 | 0.21 | 0.98 – 3.15 | | 3.81 | **<0.001** | 47.00 | |
| age | -0.01 | 0.01 | -0.16 | 0.16 | -0.04 – 0.01 | | -1.01 | 0.319 | 47.00 | |
| Male | 0.39 | 0.35 | 0.16 | 0.14 | -0.32 – 1.09 | | 1.11 | 0.275 | 47.00 | |
| Other | -0.32 | 0.93 | -0.05 | 0.14 | -2.19 – 1.55 | | -0.34 | 0.732 | 47.00 | |
| YesCollegeDegree | 0.14 | 0.47 | 0.05 | 0.16 | -0.81 – 1.08 | | 0.29 | 0.774 | 47.00 | |
| GroupID [LKM] | 0.47 | 0.35 | 0.38 | 0.29 | -0.24 – 1.17 | | 1.33 | 0.189 | 47.00 | |
| stateEmp painUnpleasant | -0.06 | 0.19 | -0.04 | 0.14 | -0.44 – 0.33 | | -0.30 | 0.768 | 47.00 | |
| Observations | 54 | | | | | | | | |  |
| R^2^ / R^2^ adjusted | 0.097 / -0.018 | | | | |  | | | | |
| AIC | 185.021 | | | | | | | | |  |

*Note*. Linear regression showing the effect of state empathy (unpleasant) on inclusion of other in the self for the study partner (IOS; Aron et al., 1992).

### ***Frequentist Linear Regressions State Empathy (Pain)***

**Table 26**

Loneliness ~ stateEmp_pain + GroupID + age + Male + Other + YesCollegeDegree

| *Predictors* | *b* | | *SE* | | *std. B* | | *std. SE* | | *95% CI* | | | *t* | | *p* | | *df* | |
| --- | --- | --- | --- | --- | --- | --- | --- | --- | --- | --- | --- | --- | --- | --- | --- | --- | --- |
| (Intercept) | 2.24 | 0.20 | | -0.26 | | 0.20 | | 1.84 – 2.64 | | | 11.23 | | **<0.001** | | 47.00 | | |
| age | -0.00 | 0.00 | | -0.11 | | 0.15 | | -0.01 – 0.01 | | | -0.72 | | 0.476 | | 47.00 | | |
| Male | -0.13 | 0.12 | | -0.15 | | 0.14 | | -0.37 – 0.11 | | | -1.11 | | 0.274 | | 47.00 | | |
| Other | 0.40 | 0.32 | | 0.18 | | 0.14 | | -0.24 – 1.03 | | | 1.26 | | 0.214 | | 47.00 | | |
| YesCollegeDegree | -0.09 | 0.16 | | -0.09 | | 0.16 | | -0.41 – 0.23 | | | -0.55 | | 0.583 | | 47.00 | | |
| GroupID [LKM] | 0.21 | 0.12 | | 0.49 | | 0.28 | | -0.03 – 0.45 | | | 1.74 | | 0.088 | | 47.00 | | |
| stateEmp painRating | -0.02 | 0.07 | | -0.04 | | 0.14 | | -0.15 – 0.11 | | | -0.31 | | 0.757 | | 47.00 | | |
| Observations | 54 | | | | | | | | | | | | | | | |  |
| R^2^ / R^2^ adjusted | 0.149 / 0.041 | | | | | | | | |  | | | | | | | |
| AIC | 68.043 | | | | | | | | | | | | | | | |  |

*Note*. Linear regression showing the effect of state empathy (pain) on trait loneliness (UCLA; Russell, 1996).

**Table 27**

Social Connectedness ~ stateEmp_pain + GroupID + age + Male + Other + YesCollegeDegree

| *Predictors* | *b* | | *SE* | | *std. B* | | *std. SE* | | *95% CI* | | *t* | *p* | | *df* | | |
| --- | --- | --- | --- | --- | --- | --- | --- | --- | --- | --- | --- | --- | --- | --- | --- | --- |
| (Intercept) | 4.55 | 0.37 | | 0.12 | | 0.21 | | 3.82 – 5.29 | | | 12.45 | **<0.001** | 47.00 | | |  |
| age | 0.00 | 0.01 | | 0.05 | | 0.16 | | -0.01 – 0.02 | | | 0.34 | 0.738 | 47.00 | | |  |
| Male | 0.06 | 0.22 | | 0.04 | | 0.15 | | -0.38 – 0.49 | | | 0.25 | 0.801 | 47.00 | | |  |
| Other | -0.53 | 0.58 | | -0.14 | | 0.15 | | -1.70 – 0.63 | | | -0.92 | 0.364 | 47.00 | | |  |
| YesCollegeDegree | -0.13 | 0.29 | | -0.07 | | 0.16 | | -0.71 – 0.45 | | | -0.45 | 0.655 | 47.00 | | |  |
| GroupID [LKM] | -0.16 | 0.22 | | -0.22 | | 0.30 | | -0.61 – 0.28 | | | -0.74 | 0.464 | 47.00 | | |  |
| stateEmp painRating | 0.04 | 0.12 | | 0.04 | | 0.15 | | -0.21 – 0.28 | | | 0.29 | 0.775 | 47.00 | | |  |
| Observations | 54 | | | | | | | | | | | | | |  | |
| R^2^ / R^2^ adjusted | 0.041 / -0.082 | | | | | | | | |  | | | | | | |
| AIC | 133.415 | | | | | | | | | | | | | |  | |

*Note*. Linear regression showing the effect of state empathy (pain) on trait social connectedness (SCS-R; Lee et al., 2001).

**Table 28**

Trait Empathic Concern (IRI) ~ stateEmp_pain + GroupID + age + Male + Other + YesCollegeDegree

| *Predictors* | *b* | *SE* | *std. B* | *std. SE* | *95% CI* | | *t* | *p* | *df* | |
| --- | --- | --- | --- | --- | --- | --- | --- | --- | --- | --- |
| (Intercept) | 4.37 | 0.28 | 0.13 | 0.21 | 3.80 – 4.94 | | 15.46 | **<0.001** | 47.00 | |
| age | -0.00 | 0.01 | -0.00 | 0.16 | -0.01 – 0.01 | | -0.02 | 0.983 | 47.00 | |
| Male | -0.30 | 0.17 | -0.26 | 0.14 | -0.64 – 0.04 | | -1.80 | 0.078 | 47.00 | |
| Other | -0.49 | 0.45 | -0.16 | 0.14 | -1.39 – 0.41 | | -1.10 | 0.278 | 47.00 | |
| YesCollegeDegree | 0.02 | 0.22 | 0.01 | 0.16 | -0.43 – 0.47 | | 0.09 | 0.931 | 47.00 | |
| GroupID [LKM] | -0.14 | 0.17 | -0.24 | 0.29 | -0.49 – 0.20 | | -0.83 | 0.410 | 47.00 | |
| stateEmp painRating | 0.05 | 0.09 | 0.08 | 0.14 | -0.14 – 0.24 | | 0.53 | 0.599 | 47.00 | |
| Observations | 54 | | | | | | | | |  |
| R^2^ / R^2^ adjusted | 0.082 / -0.035 | | | | |  | | | | |
| AIC | 119.774 | | | | | | | | |  |

*Note*. Linear regression showing the effect of state empathy (pain) on trait empathic concern (IRI; Davis, 1983).

**Table 29**

Trait Perspective Taking (IRI) ~ stateEmp_pain + GroupID + age + Male + Other + YesCollegeDegree

| *Predictors* | *b* | *SE* | *std. B* | *std. SE* | *95% CI* | | *t* | *p* | *df* | |
| --- | --- | --- | --- | --- | --- | --- | --- | --- | --- | --- |
| (Intercept) | 4.29 | 0.32 | 0.09 | 0.21 | 3.65 – 4.94 | | 13.32 | **<0.001** | 47.00 | |
| age | -0.01 | 0.01 | -0.28 | 0.16 | -0.03 – 0.00 | | -1.80 | 0.078 | 47.00 | |
| Male | 0.03 | 0.19 | 0.02 | 0.15 | -0.36 – 0.42 | | 0.16 | 0.871 | 47.00 | |
| Other | -0.07 | 0.51 | -0.02 | 0.15 | -1.10 – 0.96 | | -0.13 | 0.894 | 47.00 | |
| YesCollegeDegree | 0.16 | 0.26 | 0.10 | 0.16 | -0.35 – 0.68 | | 0.63 | 0.531 | 47.00 | |
| GroupID [LKM] | -0.11 | 0.20 | -0.17 | 0.29 | -0.50 – 0.28 | | -0.57 | 0.568 | 47.00 | |
| stateEmp painRating | 0.09 | 0.11 | 0.13 | 0.15 | -0.12 – 0.31 | | 0.88 | 0.385 | 47.00 | |
| Observations | 54 | | | | | | | | |  |
| R^2^ / R^2^ adjusted | 0.070 / -0.048 | | | | |  | | | | |
| AIC | 120.481 | | | | | | | | |  |

*Note*. Linear regression showing the effect of state empathy (pain) on trait perspective taking (IRI; Davis, 1983).

**Table 30**

*Mean IOS ~ stateEmp_pain + GroupID + age +* Male + Other + YesCollegeDegree

| *Predictors* | *b* | *SE* | *std. B* | *std. SE* | *95% CI* | | *t* | *p* | *df* | |
| --- | --- | --- | --- | --- | --- | --- | --- | --- | --- | --- |
| (Intercept) | 3.53 | 0.41 | 0.21 | 0.20 | 2.72 – 4.35 | | 8.71 | **<0.001** | 47.00 | |
| age | -0.02 | 0.01 | -0.28 | 0.15 | -0.04 – 0.00 | | -1.88 | 0.067 | 47.00 | |
| Male | 0.54 | 0.24 | 0.31 | 0.14 | 0.06 – 1.03 | | 2.24 | **0.030** | 47.00 | |
| Other | 1.09 | 0.64 | 0.23 | 0.14 | -0.21 – 2.38 | | 1.69 | 0.097 | 47.00 | |
| YesCollegeDegree | 0.04 | 0.32 | 0.02 | 0.15 | -0.60 – 0.69 | | 0.14 | 0.890 | 47.00 | |
| GroupID [LKM] | -0.35 | 0.25 | -0.39 | 0.27 | -0.84 – 0.15 | | -1.41 | 0.166 | 47.00 | |
| stateEmp painRating | -0.13 | 0.14 | -0.13 | 0.14 | -0.40 – 0.14 | | -0.96 | 0.343 | 47.00 | |
| Observations | 54 | | | | | | | | |  |
| R^2^ / R^2^ adjusted | 0.193 / 0.091 | | | | |  | | | | |
| AIC | 144.540 | | | | | | | | |  |

*Note*. Linear regression showing the effect of state empathy (pain) on mean inclusion of other in the self (IOS; Aron et al., 1992).

**Table 31**

IOS Stranger ~ stateEmp_pain + GroupID + age + Male + Other + YesCollegeDegree

| *Predictors* | *b* | *SE* | *std. B* | *std. SE* | *95% CI* | | *t* | *p* | *df* | |
| --- | --- | --- | --- | --- | --- | --- | --- | --- | --- | --- |
| (Intercept) | 1.82 | 0.36 | 0.04 | 0.19 | 1.10 – 2.54 | | 5.06 | **<0.001** | 47.00 | |
| age | -0.02 | 0.01 | -0.35 | 0.14 | -0.04 – -0.00 | | -2.47 | **0.017** | 47.00 | |
| Male | 0.62 | 0.21 | 0.38 | 0.13 | 0.19 – 1.05 | | 2.89 | **0.006** | 47.00 | |
| Other | 0.31 | 0.57 | 0.07 | 0.13 | -0.83 – 1.46 | | 0.55 | 0.585 | 47.00 | |
| YesCollegeDegree | 0.57 | 0.29 | 0.29 | 0.15 | -0.01 – 1.14 | | 1.99 | 0.052 | 47.00 | |
| GroupID [LKM] | -0.06 | 0.22 | -0.07 | 0.27 | -0.50 – 0.38 | | -0.26 | 0.793 | 47.00 | |
| stateEmp painRating | 0.06 | 0.12 | 0.07 | 0.13 | -0.18 – 0.30 | | 0.51 | 0.612 | 47.00 | |
| Observations | 54 | | | | | | | | |  |
| R^2^ / R^2^ adjusted | 0.246 / 0.150 | | | | |  | | | | |
| AIC | 131.543 | | | | | | | | |  |

*Note*. Linear regression showing the effect of state empathy (pain) on inclusion of other in the self for a stranger (IOS; Aron et al., 1992).

**Table 32**

IOS Study Partner ~ stateEmp_pain + GroupID + age + Male + Other + YesCollegeDegree

| *Predictors* | *b* | *SE* | *std. B* | *std. SE* | *95% CI* | | *t* | *p* | *df* | |
| --- | --- | --- | --- | --- | --- | --- | --- | --- | --- | --- |
| (Intercept) | 2.33 | 0.59 | -0.23 | 0.21 | 1.15 – 3.51 | | 3.98 | **<0.001** | 47.00 | |
| age | -0.01 | 0.01 | -0.17 | 0.16 | -0.04 – 0.01 | | -1.07 | 0.289 | 47.00 | |
| Male | 0.35 | 0.35 | 0.14 | 0.14 | -0.35 – 1.06 | | 1.01 | 0.320 | 47.00 | |
| Other | -0.48 | 0.93 | -0.07 | 0.14 | -2.34 – 1.39 | | -0.51 | 0.610 | 47.00 | |
| YesCollegeDegree | 0.15 | 0.46 | 0.05 | 0.16 | -0.79 – 1.08 | | 0.31 | 0.756 | 47.00 | |
| GroupID [LKM] | 0.54 | 0.35 | 0.44 | 0.29 | -0.18 – 1.25 | | 1.52 | 0.136 | 47.00 | |
| stateEmp painRating | 0.18 | 0.20 | 0.13 | 0.14 | -0.22 – 0.57 | | 0.90 | 0.371 | 47.00 | |
| Observations | 54 | | | | | | | | |  |
| R^2^ / R^2^ adjusted | 0.111 / -0.003 | | | | |  | | | | |
| AIC | 184.194 | | | | | | | | |  |

*Note*. Linear regression showing the effect of state empathy (pain) on inclusion of other in the self for the study partner (IOS; Aron et al., 1992).

### ***Bayes Factors for Null Findings: Linear Regressions State Empathy***

To formally evaluate evidence for the absence of effects, we again computed and evaluated Bayes factors (BFs) for select predictors converting relevant *t-statistic*s using the same approach in R as outlined above.

**Table 33**

*Null Findings: Effect* of state empathy (fear) on several outcomes

| *Outcome (Null Finding)* | *b* | *t* | *p* | *BF_10_* | *BF_01_* | *Evidence* |
| --- | --- | --- | --- | --- | --- | --- |
| IRI EC | -0.25 | 6.544 | .153 | 0.153 | 6.544 | Moderate evidence for H₀ |
| IRI PT | -0.29 | 6.481 | .154 | 0.154 | 6.481 | Moderate evidence for H₀ |
| IOS | 1.78 | 1.559 | .642 | 0.642 | 1.559 | Anecdotal evidence for H₀ |
| IOS Stranger | 1.81 | 1.464 | .683 | 0.683 | 1.464 | Anecdotal evidence for H₀ |
| IOS Study Partner | 0.97 | 4.313 | .232 | 0.232 | 4.313 | Moderate evidence for H₀ |

*Note*. Trait Empathy measured using IRI with subscales Empathic Concern (EC), Perspective Taking (PT). Inclusion of Other in the Self was measured toward others on average (IOS), toward strangers (IOS Stranger), and toward the Study Partner (IOS Study Partner). All measures were taken at T2 (after the intervention). Bayes Factors based on t-statistics provide moderate evidence in support for the null hypothesis for all outcomes except mean IOS and IOS Stranger. This suggests that state empathy for fearful anticipation likely has no effect on trait empathy, nor on inclusion of other in the self with the study partner, while we can make no conclusive judgments regarding IOS and IOS stranger at this point.

**Table 34**

*Null Findings: Effect of state empath*y (unpleasantness) on several outcomes

| *Outcome (Null Finding)* | *b* | *t* | *p* | *BF_10_* | *BF_01_* | *Evidence* |
| --- | --- | --- | --- | --- | --- | --- |
| IRI EC | 0.94 | 4.437 | .225 | 0.225 | 4.437 | Moderate evidence for H₀ |
| IRI PT | 0.38 | 6.279 | .159 | 0.159 | 6.279 | Moderate evidence for H₀ |
| SCS-R | 1.87 | 1.346 | .743 | 0.743 | 1.346 | Anecdotal evidence for H₀ |
| IOS | -0.07 | 6.724 | .149 | 0.149 | 6.724 | Moderate evidence for H₀ |
| IOS Stranger | 0.75 | 5.161 | .194 | 0.194 | 5.161 | Moderate evidence for H₀ |
| IOS Study Partner | -0.30 | 6.462 | .155 | 0.155 | 6.462 | Moderate evidence for H₀ |

*Note*. Trait Empathy measured using IRI with subscales Empathic Concern (EC), Perspective Taking (PT). Social Connectedness (SCS-R) and Loneliness (UCLA) were assessed. Inclusion of Other in the Self was measured toward others on average (IOS), toward strangers (IOS Stranger), and toward the Study Partner (IOS Study Partner). Confirming the frequentist analysis, we found moderate support for the null hypothesis for all tested outcomes, indicating that state empathy for unpleasantness was unrelated to trait empathy and IOS. For social connectedness, we found anecdotal evidence for the null hypothesis so interpretation for this effect remains inconclusive.

**Table 35**

Null Findings: Effect of state empathy (pain) on several outcomes

| *Outcome (Null Finding)* | *b* | *t* | *p* | *BF_10_* | *BF_01_* | *Evidence* |
| --- | --- | --- | --- | --- | --- | --- |
| IRI EC | 0.53 | 5.896 | .170 | 0.170 | 5.896 | Moderate evidence for H₀ |
| IRI PT | 0.88 | 4.679 | .214 | 0.214 | 4.679 | Moderate evidence for H₀ |
| SCS-R | 0.29 | 6.477 | .154 | 0.154 | 6.477 | Moderate evidence for H₀ |
| UCLA | -0.31 | 6.435 | .155 | 0.155 | 6.435 | Moderate evidence for H₀ |
| IOS | -0.96 | 4.363 | .229 | 0.229 | 4.363 | Moderate evidence for H₀ |
| IOS Stranger | 0.51 | 5.952 | .168 | 0.168 | 5.952 | Moderate evidence for H₀ |
| IOS Study Partner | 0.90 | 4.581 | .218 | 0.218 | 4.581 | Moderate evidence for H₀ |

*Note*. Trait Empathy measured using IRI with subscales Empathic Concern (EC), Perspective Taking (PT). Social Connectedness (SCS-R) and Loneliness (UCLA) were assessed. Inclusion of Other in the Self was measured toward others on average (IOS), toward strangers (IOS Stranger), and toward the Study Partner (IOS Study Partner). Confirming the frequentist analysis, we found moderate support for the null hypothesis for all tested outcomes, indicating that state empathy for pain was unrelated to trait empathy, loneliness, and IOS.

### ***Post-hoc Sensitivity Analysis: Linear Regressions State Empathy***

To evaluate whether our study was adequately powered to detect theoretically meaningful effects, we conducted an a priori power analysis using G*Power (Faul et al., 2007). Based on prior research showing small-to-medium-to-large effects of empathy on loneliness (Beadle et al., 2012; Hu et al., 2020), and following conventional guidelines (Cohen, 1988), we defined a medium effect size of f² = 0.15 as our smallest effect size of interest (SESOI). This corresponds roughly to Cohen’s d ≈ 0.39, a magnitude typically considered small-to-moderate. Importantly, this threshold is consistent with the average strength of trait–state empathy relationships observed in recent large-scale experience sampling work. For example, Depow and Inzlicht (2025) found that trait empathy measures predicted real-world empathic states with correlations ranging from r = .11 to .37, with most effects falling within the small-to-medium range and total variance explained rarely exceeding 7–15%. Considering this, detecting effects around f² = 0.15 represents a realistic and theoretically grounded SESOI.

A power analysis indicated that a total sample size of N = 68 would be required to detect an effect of f² = 0.15 (d ≈ 0.39) with 80% power and α = .05 in a linear regression model with two predictors. Since our final sample comprised N = 54, we did not meet this target. To assess the implications of our actual sample size, we conducted a sensitivity analysis using G*Power (Faul et al., 2007), which revealed that we were powered to detect effects of f² = 0.189 (d ≈ 0.45) or larger. A post hoc power analysis indicated that, given our actual sample size, power to detect a medium effect (f² = 0.15, d ≈ 0.39) was approximately 69.6%. This is slightly below our SESOI and power goal and suggests that our study was marginally underpowered to detect small-to-medium-sized effects with high confidence. However, past work suggests that at least trait and behavioral measures of empathy often show weak or no associations (Murphy & Lilienfeld, 2019), making it less surprising if we observed nonsignificant effects between these domains.

Importantly, however, Bayesian analyses in our study provided mostly moderate support for the null hypothesis in the key models on which we base our main conclusions. This strengthens our confidence that the absence of significant effects in those cases likely reflects true absence rather than a lack of power.

## **Supplemental Information E: Neural Empathy is Linked to Self-reported Empathy but Not Loneliness**

### ***Whole Brain Results***

To assess multi-voxel pattern similarity of pain and fearful anticipation throughout the whole brain, we conducted one-sample t-tests (Table 36-38). We also conducted two-sample-test using AFNI’s 3dttest++ but there was no credible evidence for significant clusters.

**Table 36**

*Pattern similarity (Pain) Whole Brain*

| *Voxel Nr* | *Volume (mm^3^)* | *Peak x* | *Peak y* | *Peak z* | *z* | *Mean* | *SEM* | *Region* |
| --- | --- | --- | --- | --- | --- | --- | --- | --- |
| 15230 | 411210 | -59.5 | 27.5 | 44.5 | 4.58 | 0.23 | 0.001 | Multiple Regions* |
| 40 | 1080 | -2.5 | -59.5 | 29.5 | 3.60 | 0.13 | 0.004 | dACC |

*Note.* *See Table 37 for local maxima of the large cluster. Two clusters were found based on FWER (2 Nearest Neighbors (NN); p < 0.05_corr_ with underlying voxel height threshold p < 0.001). We assessed pattern similarity in the whole brain using one-sample *t*-tests employing AFNI’s 3dttest++ function. We found a large significant region indicating distributed self-other mapping across the whole brain, including regions listed in Table 4, as well as another cluster in the dACC. We used AFNI’s whereami function (MNI_Glasser_HCP_v1.0) to determine regions (Glasser et al., 2016).

**Table 37**

Local Maxima Pattern Similarity (Pain) Whole Brain

| *Index* | *Intensity* | *Peak x* | *Peak y* | *Peak z* | *Dist(mm)* | *Region* |
| --- | --- | --- | --- | --- | --- | --- |
| 1 | 0.66 | 54 | -68 | -8 | 53.833 | Right PH, V4t, PHT, FST, |
| 2 | 0.658 | 63 | -33 | 37 | 44.193 | Right PF Complex, PeriSylvian Language Area |
| 3 | 0.622 | -49 | -78 | -2 | 35.496 | Left Lateral Occipital Area 2, V4t, Posterior Infero Temporal, FST, PHT, Fusiform Complex |
| 4 | 0.537 | -61 | -27 | 44 | 20.347 | Left PF Complex, Area 1 and 2 |
| 5 | 0.418 | -58 | -45 | 33 | 20.347 | Left PF Complex, Perisylvian Language Area, PFm Complex |
| 6 | 0.254 | 45 | -1 | 9 | 44.193 | Right Frontal Opercular Area 1, 2, 3, Middle Insular Area, OP2-3/VS, OP4/PV |
| 7 | 0.252 | -43 | -4 | 12 | 40.804 | Left Frontal Opercular Area 1, 2, 4, Middle and Posterior Insula, Insular Granular Complex, OP2-3/VS |
| 8 | 0.251 | -1 | -3 | 45 | 31.032 | Left Area Posterior 24 Prime, Dorsal Area 24d, Right Posterior Area 24 prime, Right Dorsal and Ventral Area 24d, Right Area 23c, Left Area 23d |
| 9 | 0.245 | -19 | -83 | -23 | 35.496 | Left 2nd and 3rd Visual Area |
| 10 | 0.212 | 15 | -31 | 47 | 31.032 | Right Area 5m Ventral, Area 23c, Dorsal Area 24d, Area 23d |

*Note.* To identify which regions specifically were involved in whole brain decoding, we extracted local maxima within an 8 mm radius. We used AFNI’s whereami function (MNI_Glasser_HCP_v1.0; Glasser et al., 2016) to determine regions within 0-7 mm proximity. Cluster 6-10 contain the insular and cingulate cortex. MNI space coordinates are reported.

**Table 38**

Pattern similarity (Fearful Anticipation) Whole Brain

| *Voxel Nr* | *Volume (mm^3^)* | *Peak x* | *Peak y* | *Peak z* | *z* | *Mean* | *SEM* | *Region* |
| --- | --- | --- | --- | --- | --- | --- | --- | --- |
| 363 | 9801 | 33.5 | 90.5 | 3.5 | 3.85 | 0.16 | 0.002 | Left Second, Third, Fourth Visual Area, Posterior Infero Temporal |
| 91 | 2457 | 3.5 | 66.5 | 47.5 | 4.01 | 0.21 | 0.004 | Left and Right Medial Area 7P, Left and Right Parieto-Occipital Sulcus Area 2 |
| 86 | 2322 | -38.5 | -32.5 | -3.5 | 3.81 | 0.13 | 0.003 | Right Area Frontal Opercular, Ventral Insular Area, Area 54, 47I, IFSa |
| 60 | 1620 | 48.5 | -2.5 | 47.5 | 3.62 | 0.12 | 0.002 | Left Area 55b, Frontal and Premotor Eye Fields |
| 35 | 945 | -32.5 | 93.5 | 5.5 | 3.57 | 0.15 | 0.001 | Right Second, Third, and Primary Visual Area |

*Note.* Five clusters were found based on FWER (2 Nearest Neighbors (NN); *p* < .05_corr_ with underlying voxel height threshold *p* < .001). We found significant regions across the whole brain during fearful anticipation of pain. We used AFNI’s whereami function (MNI_Glasser_HCP_v1.0; Glasser et al., 2016) to determine regions within 0-7 mm proximity. Cluster 3 contains the insular cortex.

### ***Bayes Factors for Null Findings: Group Differences in Neural Empathy***

No significant clusters were detected using AFNI’s 3dttest++ function, which was used to conduct a two-sample *t*-test with permutation-based cluster-thresholding (FWER corrected *p* < 0.001) in regions of interest (dACC, left AI, right AI) and the whole brain. To formally assess the strength of evidence for null effects in each region, we again computed Bayes Factors (BFs) by rerunning the *t*-tests for the LKM versus control extracting the average *t*-statistic per region. Unlike p-values, Bayes Factors do not require multiple comparison correction, as they quantify the relative evidence for the null versus the alternative hypothesis directly (Wagenmakers et al., 2018). BFs were computed and evaluated using the previously mentioned approach in R. Across all regions, we observed moderate evidence in favor of the null hypothesis, indicating no reliable differences between participants in the LKM and PMR groups.

**Table 39**

Pattern Similarity (Pain)

| *Outcome (Null Finding)* | *t* | *df* | *BF_10_* | *BF_01_* | *Evidence* |
| --- | --- | --- | --- | --- | --- |
| dACC | 0.070 | 52 | 0.149 | 6.722 | Moderate evidence for H₀ |
| Left AI | -0.488 | 52 | 0.166 | 6.015 | Moderate evidence for H₀ |
| Right AI | -0.343 | 52 | 0.157 | 6.370 | Moderate evidence for H₀ |
| Whole Brain | 0.033 | 52 | 0.149 | 6.734 | Moderate evidence for H₀ |

*Note.* Bayes Factors support the conclusion that pattern similarity of pain is similar for LKM and PMR groups in any of our regions of interest.

**Table 40**

Pattern Similarity (Fearful Anticipation)

| *Outcome (Null Finding)* | *t* | *df* | *BF_10_* | *BF_01_* | *Evidence* |
| --- | --- | --- | --- | --- | --- |
| dACC | 0.102 | 52 | 0.149 | 6.704 | Moderate evidence for H₀ |
| Left AI | 0.550 | 52 | 0.171 | 5.834 | Moderate evidence for H₀ |
| Right AI | 0.609 | 52 | 0.177 | 5.647 | Moderate evidence for H₀ |
| Whole Brain | 0.222 | 52 | 0.152 | 6.581 | Moderate evidence for H₀ |

*Note.* Bayes Factors support the conclusion that pattern similarity of pain is similar for LKM and PMR groups in any of our regions of interest.

Moreover, we used AFNI’s 3dMVM to run several ANOVAs, testing the effect of loneliness, trait empathy (empathic concern and perspective taking), and state empathy on pattern similarity of pain and fearful anticipation separately. We extracted the average t-statistic from the 3dMVM maps for each null effect of interest and then converted these into Bayes Factors using the same approach in R as outlined above. While we found empathic concern, as well as state empathy to be associated with pattern similarity (see Results), we did not find credible evidence for main effects of loneliness or perspective taking and neither of interactions between loneliness x group nor perspective taking x group on pattern similarity. Below, we provide Bayes Factors for these null effects, providing moderate evidence to support the hypotheses that loneliness, as well as perspective-taking have no or little effect on pattern similarity.

**Table 41**

*Pattern similarity (pain) ~ Loneliness * Group*

| Region | Effect | t (50) | *BF_10_* | *BF_01_* | Evidence |
| --- | --- | --- | --- | --- | --- |
| dACC | Main Effect Group | 0.102 | 0.153 | 6.528 | Moderate for H₀ |
| dACC | Main Effect Loneliness | -0.144 | 0.154 | 6.496 | Moderate for H₀ |
| dACC | Interaction | 0.18 | 0.155 | 6.459 | Moderate for H₀ |
| Left AI | Main Effect Group | -0.567 | 0.178 | 5.632 | Moderate for H₀ |
| Left AI | Main Effect Loneliness | 0.477 | 0.17 | 5.888 | Moderate for H₀ |
| Left AI | Interaction | 0.844 | 0.214 | 4.683 | Moderate for H₀ |
| Right AI | Main Effect Group | -0.158 | 0.154 | 6.482 | Moderate for H₀ |
| Right AI | Main Effect Loneliness | -0.812 | 0.208 | 4.802 | Moderate for H₀ |
| Right AI | Interaction | 0.222 | 0.156 | 6.408 | Moderate for H₀ |

*Note*. Moderate evidence for absence of effect of loneliness on pattern similarity of pain.

**Table 42**

*Pattern similarity (fearful anticipation) ~ Loneliness * Group*

| Region | Main Effect Group | t | *BF_10_* | *BF_01_* | Evidence |
| --- | --- | --- | --- | --- | --- |
| dACC | Main Effect Loneliness | 0.065 | 0.153 | 6.546 | Moderate for H₀ |
| dACC | Interaction | 0.145 | 0.154 | 6.495 | Moderate for H₀ |
| dACC | Main Effect Group | -0.208 | 0.156 | 6.426 | Moderate for H₀ |
| Left AI | Main Effect Loneliness | 0.402 | 0.165 | 6.075 | Moderate for H₀ |
| Left AI | Interaction | 0.652 | 0.186 | 5.364 | Moderate for H₀ |
| Left AI | Main Effect Group | 0.203 | 0.155 | 6.432 | Moderate for H₀ |
| Right AI | Main Effect Loneliness | 0.625 | 0.184 | 5.449 | Moderate for H₀ |
| Right AI | Interaction | -0.19 | 0.155 | 6.448 | Moderate for H₀ |
| Right AI | Main Effect Group | -0.665 | 0.188 | 5.318 | Moderate for H₀ |

*Note*. Moderate evidence for absence of effect of loneliness on pattern similarity of fearful anticipation of pain.

**Table 43**

*Pattern similarity (pain) ~ Perspective Taking * Group*

| Region | Effect | t | *BF_10_* | *BF_01_* | Evidence |
| --- | --- | --- | --- | --- | --- |
| dACC | Main Effect Group | 0.125 | 0.154 | 6.511 | Moderate for H₀ |
| dACC | Main Effect PT | 0.584 | 0.179 | 5.579 | Moderate for H₀ |
| dACC | Interaction | 0.766 | 0.201 | 4.968 | Moderate for H₀ |
| Left AI | Main Effect Group | -0.468 | 0.169 | 5.91 | Moderate for H₀ |
| Left AI | Main Effect PT | 0.191 | 0.155 | 6.447 | Moderate for H₀ |
| Left AI | Interaction | 0.383 | 0.163 | 6.117 | Moderate for H₀ |
| Right AI | Main Effect Group | -0.282 | 0.158 | 6.316 | Moderate for H₀ |
| Right AI | Main Effect PT | 0.673 | 0.189 | 5.293 | Moderate for H₀ |
| Right AI | Interaction | 0.392 | 0.164 | 6.099 | Moderate for H₀ |

*Note*. Moderate evidence for absence of effect of perspective taking on pattern similarity of pain.

**Table 44**

*Pattern similarity (fearful anticipation) ~ Perspective Taking * Group*

| Region | Effect | t | *BF_10_* | *BF_01_* | Evidence |
| --- | --- | --- | --- | --- | --- |
| dACC | Main Effect Group | 0.059 | 0.153 | 6.549 | Moderate for H₀ |
| dACC | Main Effect PT | -0.45 | 0.168 | 5.958 | Moderate for H₀ |
| dACC | Interaction | 0.187 | 0.155 | 6.451 | Moderate for H₀ |
| Left AI | Main Effect Group | 0.499 | 0.172 | 5.827 | Moderate for H₀ |
| Left AI | Main Effect PT | -0.454 | 0.168 | 5.947 | Moderate for H₀ |
| Left AI | Interaction | 0.552 | 0.176 | 5.675 | Moderate for H₀ |
| Right AI | Main Effect Group | 0.635 | 0.185 | 5.418 | Moderate for H₀ |
| Right AI | Main Effect PT | 0.316 | 0.16 | 6.256 | Moderate for H₀ |
| Right AI | Interaction | 1.029 | 0.251 | 3.981 | Moderate for H₀ |

*Note*. Moderate evidence for absence of effect of perspective taking on pattern similarity of fearful anticipation of pain.

### ***Post-hoc Sensitivity Analysis: Group Differences in Neural Empathy***

To ensure our study was adequately powered to detect theoretically meaningful effects, we conducted power analyses using G*Power 3.1 (Faul et al., 2007). For the between-subjects comparison, we based our analysis on an independent-samples t-test with a two-tailed alpha of 0.05 and power set to 0.80. We used a medium-to-large effect size (Cohen’s *d* = 0.80), informed by prior work on neural responses to empathy-related stimuli (e.g., Mascaro et al., 2013, who reported *d* = 0.79 for right AI activity in relation to compassion meditation). This analysis indicated a required total sample size of 52 participants (26 per group). To account for our actual sample sizes (n = 25 vs. 29), we also performed a post hoc sensitivity analysis, which revealed that our design was powered to detect an only slightly smaller effect of *d* = 0.78 with 80% power. Notably, we found moderate evidence for the null hypothesis across all regions of interest, based on Bayes Factors (BFs₀₁ between 5.6 and 6.7), indicating support for the absence of differences between LKM and PMR in neural empathy-related responses (see Table 38 and 39).

For our ROI-based voxel-wise ANOVA analyses conducted with AFNI’s 3dMVM, no prior studies had tested the same hypothesis with this design. Thus, we assumed a medium effect size (*f* = 0.25) for the interaction between group and time (10 pain trials). Given our design with two groups and ten repeated measures per subject, a power analysis showed that a total of 14 participants would be sufficient to detect a medium effect at 80% power. With 54 participants included in our study, we thus substantially exceeded this minimum. A post hoc sensitivity analysis indicated that our actual design was powered to detect an effect size as small as *f* = 0.12, equivalent to Cohen’s *d* ≈ 0.24, when converted using the formula *d* = 2f. We also conducted a post hoc achieved power analysis, which indicated 99.9% power to detect an effect of f = 0.25 with our sample size and design. Importantly, power is likely underestimated in both the t-test and ANOVA analyses because G*Power does not fully account for the increased precision of fMRI data stemming from multiple measurements (i.e., trial-level or voxel-level estimates within each subject) (cf. Mumford & Nichols, 2008). Therefore, our analyses were likely more sensitive than GPower estimates suggest.

## **Supplemental Information F: Multi-Voxel Cross-Classification (MVCC) of Pain and Fearful Anticipation**

Similar to our prior work (O’Connell et al., 2019), we used machine-learning based multi-voxel cross-classification (MVCC) as an alternative approach to assessing self-other mapping of pain and fearful anticipation. AFNI’s 3dLSS least-squares sum regression (Cox, 1996) was used to obtain parameter estimates for each event (Mumford, 2012). Analyses were performed on unsmoothed parameter estimate images in The Decoding Toolbox (Hebart et al., 2015). Analyses were performed separately on the left Anterior Insula (left AI), the right Anterior Insula (right AI), and the dorsal Anterior Cingulate Cortex (dACC). A linear SVM classifier was trained on neural response patterns during one context and tested on response patterns during a different context (i.e. trained on experienced pain versus no-pain then tested on observed pain versus no-pain; trained on experienced fearful anticipation of pain versus no pain anticipation then tested on observed fearful anticipation of pain versus no anticipation). We performed each cross-classification analysis twice, bidirectionally training and testing each context and averaging the result, consistent with prior approaches (Corradi-Dell’Acqua et al., 2011; Man et al., 2012; O’Connell et al., 2019; Oosterhof et al., 2012). To evaluate classifier performance, we calculated AUC, a sensitive metric that reduces potential bias due to unbalanced classes (Jeni et al., 2013). One AUC-minus-chance value was computed for each ROI per participant and for each analysis.

While the decoding approach similarly revealed significant self-other cross-classification of pain and fearful anticipation, we decided to complete the main part of the analysis using the multi-voxel approach as outlined in the methods section because the empathic pain task has not been optimized for this type of machine-learning analysis. Specifically, more runs may be needed (not just one for self and other, all conditions should be in one run (self and other), and the classes of all stimuli would need to be balanced (there should be equal numbers of pain/fearful anticipation and no pain/fearful anticipation trials, which is not the case in our task and would extend the length of our task by a large amount) to achieve ideal sensitivity of the approach.

We assessed self-other cross-classification of pain and fearful anticipation in the whole brain using one-sample *t*-tests employing AFNI’s 3dttest++ function. We found a large significant region indicating distributed self-other mapping across the whole brain (see Table 6). To identify which regions specifically were involved, we extracted local maxima within an 8 mm radius (see Table 47). We also found significant regions across the whole brain during fearful anticipation of pain (see Table 48). We also completed a two-sample-test using AFNI’s 3dttest++ but did not find whole-brain differences in pattern similarity of pain or fear between meditation groups.

**Table 45**

AUC (Pain) AI and dACC

| *Voxel Nr* | *Volume (mm^3^)* | *Peak x* | *Peak y* | *Peak z* | *z* | *Mean* | *SEM* | *Region* |
| --- | --- | --- | --- | --- | --- | --- | --- | --- |
| 21 | 567 | 0.5 | -8.5 | 32.5 | 3.56 | 5.51 | 0.101 | dACC |
| 14 | 378 | -8.5 | -11.5 | 35.5 | 3.51 | 4.94 | 0.127 | dACC |
| 22 | 594 | 42.5 | -2.5 | -6.5 | 3.67 | 6.18 | 0.090 | Left AI |
| 16 | 432 | -41.5 | -2.5 | 2.5 | 4.16 | 7.41 | 0.084 | Right AI |

*Note.* Four clusters were found based on FWER (2 Nearest Neighbors (NN); *p* < .05_corr_ with underlying voxel height threshold *p* < .001) Region of Interest Analysis of dACC, left AI, right AI.

* No sig clusters for AUC fearful anticipation

**Table 46**

AUC (Pain) Whole Brain

| *Voxel Nr* | *Volume (mm^3^)* | *Peak x* | *Peak y* | *Peak z* | *z* | *Mean* | *SEM* | *Region* |
| --- | --- | --- | --- | --- | --- | --- | --- | --- |
| 14576 | 393552 | -50.5 | 57.5 | -0.5 | 4.59 | 8.65 | 0.027 | Right Area PH, PHT, Temporo Parietal Occipital Junction 2 |
| 374 | 10098 | -11.5 | 21.5 | 41.5 | 3.99 | 6.22 | 0.057 | Right Area 5m Ventral, Dorsal Area 24d, 23d |
| 123 | 3321 | -17.5 | 75.5 | 48.5 | 4.11 | 6.76 | 0.113 | Right Dorsal Transitional Visual Area, V6A, IPS 1, Parietal-Occipital Sulcus 2, Visual Area 6 |
| 45 | 1215 | -17.5 | 60.5 | -3.5 | 3.59 | 5.41 | 0.093 | Visual Area 2, 4, Ventromedial Visual Area 2, 3, |
| 39 | 1053 | 9.5 | 59.5 | -3.5 | 3.61 | 4.53 | 0.078 | Visual Area 1, 3, Ventromedial Visual Area 1 |

*Note.* Five clusters were found based on FWER (2 Nearest Neighbors (NN); *p* < .05_corr_ with underlying voxel height threshold *p* < .001). We used AFNI’s whereami function (MNI_Glasser_HCP_v1.0) to determine regions (Glasser et al., 2016).

**Table 47**

Local Maxima AUC (Pain) Whole Brain 2, 4, 5, 6,

| *Index* | *Intensity* | *Peak x* | *Peak z* | *Peak z* | *Dist (mm)* | *Region* |
| --- | --- | --- | --- | --- | --- | --- |
| 1 | 20.385 | 48 | -66 | -1 | 31.464 | Right FST, V4t, MST, PHT |
| 2 | 19.186 | 63 | -26 | 21 | 22.045 | Right PF Opercular, Peri Sylvian Language Area, Auditory Complex 4, Para Belt Comp  lex, Retro Insular Cortex |
| 3 | 12.619 | 42 | -43 | 53 | 17.234 | Right 7PC, PFm Complex, Intraparietal Area 2, Lateral Ventral Intraparietal Area |
| 4 | 12.601 | -43 | -4 | 12 | 48.836 | Left Frontal Opercular Area 2, Middle Insular Area, Posterior Insular Area 2, Frontal Opercular Area 1, 4, Insular Granular Complex, OP2-3/VS |
| 5 | 12.425 | 42 | -4 | 9 | 12.728 | Right Posterior Insular Area 2, Insular Granular Complex, Frontal Opercular Area 1, 3, Middle Insular Area |
| 6 | 11.719 | 45 | -7 | -5 | 12.728 | Right Posterior Insular Area, Para-insular Area, Posterior Insular Area 1, Area 52, Medial Belt Complex, anterior STSd |
| 7 | 11.045 | 36 | -59 | 58 | 17.234 | Right Area 7PC, Lateral Dorsal and Ventral IntraParietal Area, PFm Complex, Ventral Intraparietal Complex |
| 8 | 10.867 | -43 | -49 | 49 | 55.317 | Left PFm Complex, 7PC Area, Intraparietal Area 2, Anterior Intraparietal Area, Lateral Ventral Intraparietal Area |
| 9 | 10.561 | 33 | -94 | 4 | 31.464 | Right Visual Area 2, 3, 4, V3CD Area, Lateral Occipital Area 2 |
| 10 | 8.295 | 57 | -28 | -2 | 22.045 | Right Posterior STSv, Auditory Complex 5, Middle TE1 Area |
| 11 | 8.131 | -43 | 45 | 25 | 48.836 | Left Area 46, Anterior Area 9-46v, 9-46d Area |

*Note.* To identify which regions specifically were involved in whole brain decoding of pain, we extracted local maxima within an 8 mm radius. We used AFNI’s whereami function (MNI_Glasser_HCP_v1; Glasser et al., 2016) to determine regions within 0-7 mm proximity. Cluster 2, 4, 5, 6 contain the insular cortex. MNI space coordinates are reported.

**Table 48**

AUC (Fearful Anticipation) Whole Brain

| *Voxel Nr* | *Volume (mm* | *Peak x* | *Peak y* | *Peak z* | *z* | *Mean* | *SEM* | *Region* |
| --- | --- | --- | --- | --- | --- | --- | --- | --- |
| 69 | 1863 | -35.5 | -32.5 | -3.5 | 3.72 | 4.69 | 0.062 | Right Anterior Ventral Insular Area, Frontal Opercular Area, Area 47m |
| 63 | 1701 | -47.5 | 63.5 | -0.5 | 3.60 | 3.94 | 0.056 | Right PH, V4t, PHT Area, MST Area |
| 60 | 1620 | 12.5 | 102.5 | 8.5 | 3.58 | 3.74 | 0.043 | Left Visual Area 1, 2 |

*Note.* Five clusters were found based on FWER (2 Nearest Neighbors (NN); *p* < .05_corr_ with underlying voxel height threshold *p* < .001). We used AFNI’s whereami function (MNI_Glasser_HCP_v1.0) to determine regions (Glasser et al., 2016).

**Figure 4**

*Self-other Cross-Classification of Pain and Fearful Anticipation Whole Brain*


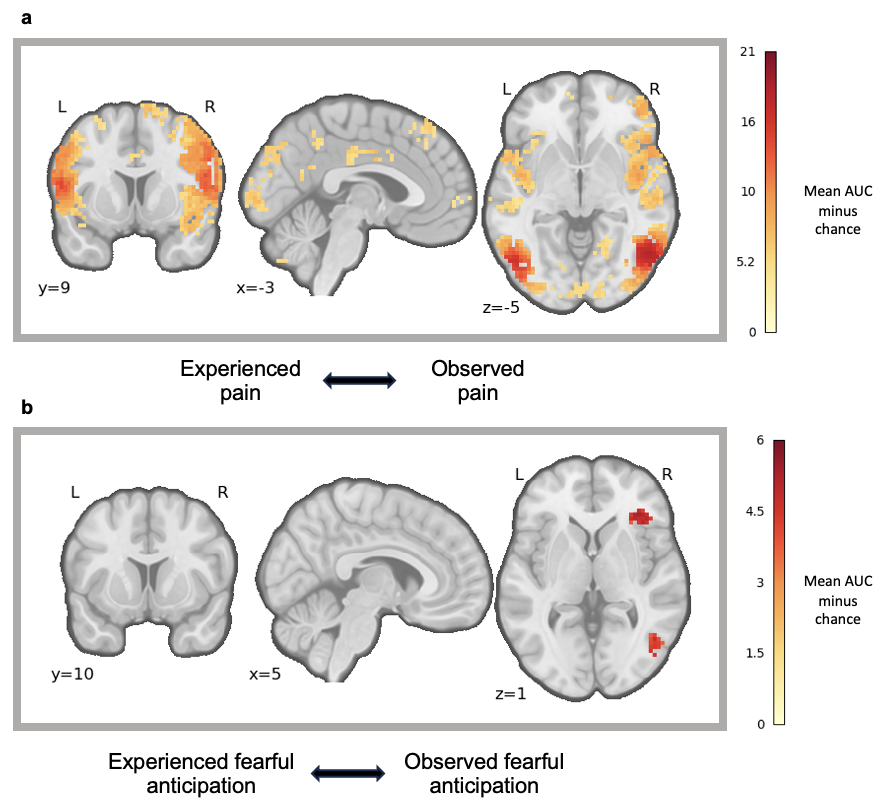


*Note.* Cross-classification (Whole Brain). (a) Both groups revealed above chance cross-classification between experienced and observed pain in regions including Right TPJ, perisylvian fissure, right intraparietal area, left and right insula, right STS, middle frontal gyrus, and ACC. (b) Both groups revealed above chance cross-classification between experienced and observed fearful anticipation of pain in regions including left visual cortex, left and right parietal-occipital sulcus, right AI, left premotor area, right visual cortex.

## **Supplemental Information G: Deviations from the Preregistration**

Several deviations from the preregistration were made to improve the analysis plan. First, we did not conduct mediation analyses as originally planned because the effect of meditation group on loneliness and empathy was not significant, eliminating the rationale for such analyses. Second, because loneliness and social connectedness were so highly correlated (*r* =-0.77***), we analyzed only loneliness in our neuroimaging analysis investigating neural empathic responding to reduce the total number of tests. We also did not include the variable love to narrow our focus and because it shows poor internal consistency and conceptual heterogeneity (Dixson et al., 2018). We also focused specifically on empathic concern and perspective taking subscales of the IRI as our primary measure of trait empathy, given these are the scales relevant prosocial outcomes. Third, we expanded our focus of neural empathic responding beyond pain to include fearful anticipation, following our previous work using this task (Brethel-Haurwitz et al., 2018; O’Connell et al., 2019), providing a more comprehensive examination of neural empathy. Additionally, we refined our terminology, referring to "shared neural representations" or “self-other neural mapping” as "neural empathy" for clarity. This construct was operationalized as multi-voxel pattern similarity of experienced and observed pain and fearful anticipation of pain. Fourth, we deviated from the preregistered plan to conduct machine-learning based cross-classification analyses as our primary methodology. Upon further investigation, we determined that the task design was not optimal for this approach. After the preregistration, we learned that MVCC generally requires balanced conditions and training/testing partitions across multiple acquisitions of runs (i.e., leave-one-run-out cross-validation) (Weaverdyck et al., 2020). Conceptually, MVCC has interpretations analogous to pattern similarity: both assess how similar vs. distinguishable multivoxel response patterns are, but MVCC expresses this via out-of-sample classification generalization rather than a direct similarity metric. This analysis was conducted using a representational dissimilarity matrix (RDM) searchlight approach, allowing for a nuanced assessment of neural empathic responding. We nonetheless report the results of the cross-classification analysis in the Supplemental Information F. Fifth, the sample sizes differed slightly from the preregistration due to participant exclusions, resulting in group sizes of 25 and 29 rather than the originally planned 27 per group. All deviations were made to ensure the most accurate and meaningful interpretations of the data while maintaining the integrity of the research objectives.

## **References**

Aron, A., Aron, E. N., & Smollan, D. (1992). Inclusion of Other in the Self Scale and the Structure of Interpersonal Closeness. *Journal of Personality and Social Psychology*, *63(4)*, 596–612. https://doi.org/10.1037/0022-3514.63.4.596

Beadle, J. N., Keady, B., Brown, V., Tranel, D., & Paradiso, S. (2012). Trait Empathy as a Predictor of Individual Differences in Perceived Loneliness. *Psychological Reports*, *110*(1), 3–15. https://doi.org/10.2466/07.09.20.PR0.110.1.3-15

Brethel-Haurwitz, K. M., Cardinale, E. M., Vekaria, K. M., Robertson, E. L., Walitt, B., VanMeter, J. W., & Marsh, A. A. (2018). Extraordinary Altruists Exhibit Enhanced Self–Other Overlap in Neural Responses to Distress. *Psychological Science*, *29*(10), 1631–1641. https://doi.org/10.1177/0956797618779590

Cohen, J. (1988). *Statistical power analysis for the behavioral sciences* (2. ed., reprint). Psychology Press.

Corradi-Dell’Acqua, C., Hofstetter, C., & Vuilleumier, P. (2011). Felt and Seen Pain Evoke the Same Local Patterns of Cortical Activity in Insular and Cingulate Cortex. *The Journal of Neuroscience*, *31*(49), 17996–18006. https://doi.org/10.1523/JNEUROSCI.2686-11.2011

Cox, R. W. (1996). AFNI: Software for Analysis and Visualization of Functional Magnetic Resonance Neuroimages. *Computers and Biomedical Research*, *29*(3), 162–173. https://doi.org/10.1006/cbmr.1996.0014

Davis, M. H. (1983). Measuring Individual DIfferences in Empathy: Evidence for a Multidimensional Approach. *Journal of Personality and Social Psychology*, *44*(1), 113–126. https://doi.org/10.1037/0022-3514.44.1.113

Depow, G. J., & Inzlicht, M. (2025). *How individual differences in empathy predict moments of empathy in everyday life*. PsyArXiv. https://doi.org/10.31234/osf.io/tbd7q_v1

Dixson, D. D., Anderson, C. L., & Keltner, D. (2018). Measuring Positive Emotions: An Examination of the Reliability and Structural Validity of Scores on the Seven Dispositional Positive Emotions Scales. *Journal of Well-Being Assessment*, *2*(2), 115–133. https://doi.org/10.1007/s41543-019-00015-y

Eklund, A., Nichols, T. E., & Knutsson, H. (2016). Cluster failure: Why fMRI inferences for spatial extent have inflated false-positive rates. *Proceedings of the National Academy of Sciences*, *113*(28), 7900–7905. https://doi.org/10.1073/pnas.1602413113

Esteban, O., Markiewicz, C. J., Blair, R. W., Moodie, C. A., Isik, A. I., Erramuzpe, A., Kent, J. D., Goncalves, M., DuPre, E., Snyder, M., Oya, H., Ghosh, S. S., Wright, J., Durnez, J., Poldrack, R. A., & Gorgolewski, K. J. (2019). fMRIPrep: A robust preprocessing pipeline for functional MRI. *Nature Methods*, *16*(1), 111–116. https://doi.org/10.1038/s41592-018-0235-4

Faul, F., Erdfelder, E., Lang, A.-G., & Buchner, A. (2007). G*Power 3: A flexible statistical power analysis program for the social, behavioral, and biomedical sciences. *Behavior Research Methods*, *39*(2), 175–191. https://doi.org/10.3758/BF03193146

Glasser, M. F., Coalson, T. S., Robinson, E. C., Hacker, C. D., Harwell, J., Yacoub, E., Ugurbil, K., Andersson, J., Beckmann, C. F., Jenkinson, M., Smith, S. M., & Van Essen, D. C. (2016). A multi-modal parcellation of human cerebral cortex. *Nature*, *536*(7615), 171–178. https://doi.org/10.1038/nature18933

Gorgolewski, K. J., Auer, T., Calhoun, V. D., Craddock, R. C., Das, S., Duff, E. P., Flandin, G., Ghosh, S. S., Glatard, T., Halchenko, Y. O., Handwerker, D. A., Hanke, M., Keator, D., Li, X., Michael, Z., Maumet, C., Nichols, B. N., Nichols, T. E., Pellman, J., … Poldrack, R. A. (2016). The brain imaging data structure, a format for organizing and describing outputs of neuroimaging experiments. *Scientific Data*, *3*(1), 160044. https://doi.org/10.1038/sdata.2016.44

Gracely, R. H., Lynch, S. A., & Bennett, G. J. (1992). Painful neuropathy: Altered central processing maintained dynamically by peripheral input. *PAIN*, *51*, 175–194. https://doi.org/10.1016/0304-3959(92)90259-E

Hebart, M. N., Görgen, K., & Haynes, J.-D. (2015). The Decoding Toolbox (TDT): A versatile software package for multivariate analyses of functional imaging data. *Frontiers in Neuroinformatics*, *8*. https://doi.org/10.3389/fninf.2014.00088

Hu, T., Zheng, X., & Huang, M. (2020). Absence and Presence of Human Interaction: The Relationship Between Loneliness and Empathy. *Frontiers in Psychology*, *11*, 768. https://doi.org/10.3389/fpsyg.2020.00768

Jeffreys, H. (1961). *Theory of probability.* (3d ed.). Clarendon Press.

Jeni, L. A., Cohn, J. F., & De La Torre, F. (2013). Facing Imbalanced Data—Recommendations for the Use of Performance Metrics. *2013 Humaine Association Conference on Affective Computing and Intelligent Interaction*, 245–251. https://doi.org/10.1109/ACII.2013.47

Judd, C. M., Kenny, D. A., & McClelland, G. H. (2001). Estimating and testing mediation and moderation in within-subject designs. *Psychological Methods*, *6*(2), 115–134. https://doi.org/10.1037/1082-989X.6.2.115

Kreplin, U., Farias, M., & Brazil, I. A. (2018). The limited prosocial effects of meditation: A systematic review and meta-analysis. *Scientific Reports*, *8*(1), 2403. https://doi.org/10.1038/s41598-018-20299-z

Lakens, D., Scheel, A. M., & Isager, P. M. (2018). *Equivalence Testing for Psychological Research: A Tutorial*. (1(2)), 259–269. https://doi.org/10.17605/OSF.IO/QAMC6

Lee, R., Draper, M., & Lee, S. (2001). Social Connectedness, Dysfunctional Interpersonal Behaviors, and Psychological Distress: Testing a Mediator Model. *Journal of Counseling Psychology*, *48*(3), 310–318. https://doi.org/10.1037/0022-0167.48.3.310

Man, K., Kaplan, J. T., Damasio, A., & Meyer, K. (2012). Sight and Sound Converge to Form Modality-Invariant Representations in Temporoparietal Cortex. *The Journal of Neuroscience*, *32*(47), 16629–16636. https://doi.org/10.1523/JNEUROSCI.2342-12.2012

Mascaro, J. S., Rilling, J. K., Tenzin Negi, L., & Raison, C. L. (2013). Compassion meditation enhances empathic accuracy and related neural activity. *Social Cognitive and Affective Neuroscience*, *8*(1), 48–55. https://doi.org/10.1093/SCAN/NSS095

Morey, R. D., & Rouder, J. N. (2011). Bayes factor approaches for testing interval null hypotheses. *Psychological Methods*, *16*(4), 406–419. https://doi.org/10.1037/a0024377

Mumford, J. A. (2012). A power calculation guide for fMRI studies. *Social Cognitive and Affective Neuroscience*, *7*(6), 738–742. https://doi.org/10.1093/scan/nss059

Mumford, J. A., & Nichols, T. E. (2008). Power calculation for group fMRI studies accounting for arbitrary design and temporal autocorrelation. *NeuroImage*, *39*(1), 261–268. https://doi.org/10.1016/j.neuroimage.2007.07.061

Murphy, B. A., & Lilienfeld, S. O. (2019). Are self-report cognitive empathy ratings valid proxies for cognitive empathy ability? Negligible meta-analytic relations with behavioral task performance. *Psychological Assessment*, *31*, 1062–1072. https://doi.org/10.1037/pas0000732

O’Connell, K., Brethel-Haurwitz, K. M., Rhoads, S. A., Cardinale, E. M., Vekaria, K. M., Robertson, E. L., Walitt, B., VanMeter, J. W., & Marsh, A. A. (2019). Increased similarity of neural responses to experienced and empathic distress in costly altruism. *Scientific Reports*, *9*(1), 10774. https://doi.org/10.1038/s41598-019-47196-3

Oosterhof, N. N., Tipper, S. P., & Downing, P. E. (2012). Visuo-motor imagery of specific manual actions: A multi-variate pattern analysis fMRI study. *NeuroImage*, *63*(1), 262–271. https://doi.org/10.1016/j.neuroimage.2012.06.045

Rouder, J. N., Speckman, P. L., Sun, D., Morey, R. D., & Iverson, G. (2009). Bayesian t tests for accepting and rejecting the null hypothesis. *Psychonomic Bulletin & Review*, *16*(2), 225–237. https://doi.org/10.3758/pbr.16.2.225

Russell, D. W. (1996). UCLA Loneliness Scale (Version 3): Reliability, Validity, and Factor Structure. *Journal of Personality Assessment*, *66*(1), 20–40. https://doi.org/10.1207/s15327752jpa6601_2

Schmalz, X., Biurrun Manresa, J., & Zhang, L. (2023). What is a Bayes factor? *Psychological Methods*, *28*(3), 705–718. https://doi.org/10.1037/met0000421

Schneider, W., Eschman, A., & Zuccolotto, A. (2002). *E-Prime (Version 2.0)* [Computer software]. Psychology Software Tools Inc. https://pstnet.com/

Teoh, S. L., Letchumanan, V., & Lee, L.-H. (2021). Can Mindfulness Help to Alleviate Loneliness? A Systematic Review and Meta-Analysis. *Frontiers in Psychology*, *12*, 633319. https://doi.org/10.3389/fpsyg.2021.633319

Tzourio-Mazoyer, N., Landeau, B., Papathanassiou, D., Crivello, F., Etard, O., Delcroix, N., Mazoyer, B., & Joliot, M. (2002). Automated Anatomical Labeling of Activations in SPM Using a Macroscopic Anatomical Parcellation of the MNI MRI Single-Subject Brain. *NeuroImage*, *15*(1), 273–289. https://doi.org/10.1006/nimg.2001.0978

Vogt, B. A. (2016). Midcingulate cortex: Structure, connections, homologies, functions and diseases. *Journal of Chemical Neuroanatomy*, *74*, 28–46. https://doi.org/10.1016/j.jchemneu.2016.01.010

Wagenmakers, E.-J., Marsman, M., Jamil, T., Ly, A., Verhagen, J., Love, J., Selker, R., Gronau, Q. F., Šmíra, M., Epskamp, S., Matzke, D., Rouder, J. N., & Morey, R. D. (2018). Bayesian inference for psychology. Part I: Theoretical advantages and practical ramifications. *Psychonomic Bulletin & Review*, *25*(1), 35–57. https://doi.org/10.3758/s13423-017-1343-3

Weaverdyck, M. E., Lieberman, M. D., & Parkinson, C. (2020). Tools of the Trade Multivoxel pattern analysis in fMRI: A practical introduction for social and affective neuroscientists. *Social Cognitive and Affective Neuroscience*, *15*(4), 487–509. https://doi.org/10.1093/scan/nsaa057

Westfall, J., Kenny, D. A., & Judd, C. M. (2014). Statistical power and optimal design in experiments in which samples of participants respond to samples of stimuli. *Journal of Experimental Psychology: General*, *143*(5), 2020–2045. https://doi.org/10.1037/xge0000014

Yarkoni, T., Poldrack, R. A., Nichols, T. E., Van Essen, D. C., & Wager, T. D. (2011). Large-scale automated synthesis of human functional neuroimaging data. *Nature Methods*, *8*(8), 665–670. https://doi.org/10.1038/nmeth.1635
